# Gene expression in whole lung and pulmonary macrophages reflects the dynamic pathology associated with airway surface dehydration

**DOI:** 10.1186/1471-2164-15-726

**Published:** 2014-09-10

**Authors:** Yogesh Saini, Hong Dang, Alessandra Livraghi-Butrico, Elizabeth J Kelly, Lisa C Jones, Wanda K O’Neal, Richard C Boucher

**Affiliations:** Marsico Lung Institute/University of North Carolina Cystic Fibrosis Center, School of Medicine, University of North Carolina at Chapel Hill, 7011 Thurston Bowles Building, Chapel Hill, NC 27599-7248 USA

**Keywords:** *Scnn1b*-Tg mice, Pulmonary macrophage activation, Inflammation, Mucus clearance defect, Gene expression profiling, Lung development, Airway surface liquid dehydration

## Abstract

**Background:**

Defects in airway mucosal defense, including decreased mucus clearance, contribute to the pathogenesis of human chronic obstructive pulmonary diseases. *Scnn1b*-Tg mice, which exhibit chronic airway surface dehydration from birth, can be used as a model to study the pathogenesis of muco-obstructive lung disease across developmental stages. To identify molecular signatures associated with obstructive lung disease in this model, gene expression analyses were performed on whole lung and purified lung macrophages collected from *Scnn1b*-Tg and wild-type (WT) littermates at four pathologically relevant time points. Macrophage gene expression at 6 weeks was evaluated in mice from a germ-free environment to understand the contribution of microbes to disease development.

**Results:**

Development- and disease-specific shifts in gene expression related to *Scnn1b* over-expression were revealed in longitudinal analyses. While the total number of transgene-related differentially expressed genes producing robust signals was relatively small in whole lung (n = 84), Gene Set Enrichment Analysis (GSEA) revealed significantly perturbed biological pathways and interactions between normal lung development and disease initiation/progression. Purified lung macrophages from *Scnn1b*-Tg mice exhibited numerous robust and dynamic gene expression changes. The expression levels of Classically-activated (M1) macrophage signatures were significantly altered at post-natal day (PND) 3 when *Scnn1b*-Tg mice lung exhibit spontaneous bacterial infections, while alternatively-activated (M2) macrophage signatures were more prominent by PND 42, producing a mixed M1-M2 activation profile. While differentially-regulated, inflammation-related genes were consistently identified in both tissues in *Scnn1b*-Tg mice, there was little overlap between tissues or across time, highlighting time- and tissue-specific responses. Macrophages purified from adult germ-free *Scnn1b*-Tg mice exhibited signatures remarkably similar to non-germ-free counterparts, indicating that the late-phase macrophage activation profile was not microbe-dependent.

**Conclusions:**

Whole lung and pulmonary macrophages respond independently and dynamically to local stresses associated with airway mucus stasis. Disease-specific responses interact with normal developmental processes, influencing the final state of disease in this model. The robust signatures observed in *Scnn1b*-Tg lung macrophages highlight their critical role in disease pathogenesis. These studies emphasize the importance of region-, cell-type-, and time-dependent analyses to fully dissect the natural history of disease and the consequences of disease on normal lung development.

**Electronic supplementary material:**

The online version of this article (doi:10.1186/1471-2164-15-726) contains supplementary material, which is available to authorized users.

## Background

Defects in mucus clearance are characteristic of muco-obstructive pulmonary diseases of genetic or environmental origins, e.g., cystic fibrosis (CF), primary ciliary dyskinesia (PCD), and the chronic bronchitic (CB) form of chronic obstructive pulmonary disease (COPD) [1]. Airway mucus clearance is a multifactorial process, integrating epithelial mucin secretion and airway surface hydration with ciliary beat, cough, and/or gas–liquid pumping [2, 3]. While airway remodeling and inflammation often accompany defective mucus clearance [1, 4], the mechanisms linking defective mucus clearance to obstructive lung disease are poorly understood. One paradigm is that defects in mucus clearance produce static mucus that provide a nidus for microbial colonization and resultant inflammatory responses [5]. While this concept is supported by the presence of pathogens in lungs of patients exhibiting defects in mucus clearance [6–8], the roles of the primary defects (airway surface liquid dehydration, dehydrated/concentrated mucus, lack of ciliary beat, decreased mucus clearance) in establishment of chronic inflammation are not fully appreciated. Additional complexity arises when the consequences of mucus obstruction are considered in the context of normal lung development and aging, i.e.*,* mucus obstruction early in life (CF, PCD) may generate long-term effects that would not occur if the obstruction occurs later (CB, COPD).

To model defective airway mucus clearance *in vivo,* transgenic mice over-expressing the epithelial sodium channel beta subunit [βENaC, encoded by the *Scnn1b (Sodium channel non-voltage-gated 1, beta subunit)* gene] in airway club cells (previously referred to as Clara cells, or known as CC10-expressing secretory cells) were generated [9]. The initiating pathophysiological defect in these mice, i.e.*,* airway surface liquid (ASL) depletion, produces mucus dehydration, reduced mucus clearance, and overt pulmonary disease characterized by airway mucus obstruction, inflammation, and spontaneous bacterial infection [9–13]. At birth, the lungs of *Scnn1b* transgenic (*Scnn1b*-Tg) mice are histologically normal. During the early neonatal period, i.e., post-natal day (PND) 3–10, significant tracheal mucus plugs develop that are associated with neonatal mortality and distal airway hypoxia [10–12]. Transient necrotic degeneration of intrapulmonary club cells is also observed around PND 3 [12]. Macrophage activation, neutrophilia, and bacterial infection are detected as early as PND 5 [13]. Importantly, because murine lungs continue to mature during early post-natal life [14], the disease processes from PND 3–10 are occurring during periods of active lung development, which has relevance for lung diseases such as bronchopulmonary dysplasia (BPD) and early childhood exposures to toxic or infectious agents, i.e.*,* smoke or viral infections, where inflammatory processes and development intersect to produce long-term, negative consequences for lung function [15–18]. After PND 10, mucus obstruction becomes more prominent in the main stem bronchi of the *Scnn1b*-Tg mice, airway inflammation becomes more modest, bacterial infection is intermittent, yet bronchoalveolar lavage (BAL) mucin content and mucin gene transcription remain elevated [12, 19]. Further, alveolar air space enlargement becomes clearly evident, and the incidence of bronchus associated lymphoid tissue (BALT) increases [10, 13].

Pulmonary macrophages carry out important gate-keeping roles in host defense [20]. As resident innate immune cells, they must remain quiescent in the healthy state, yet they must be able to respond when lung homeostasis is threatened. In health and disease, crosstalk occurs between the airway epithelium and macrophages via either receptor-mediated cellular interactions [21] or through humoral signals released by either cell types [22]. The airway epithelium is the epicenter of disease initiation in the *Scnn1b*-Tg mice, and macrophages are strategically positioned to respond to defects in airway clearance. A consistent feature of disease in the *Scnn1b*-Tg mice is the presence of morphologically activated pulmonary macrophages [13]. Macrophages are morphologically activated early (by 3 days of age), and previous work identified up-regulation of genes associated with macrophage activation, including chitinases, IL-13, and other cytokines [12]. Macrophage-derived protease, Mmp12, is critical for the development of the emphysema in *Scnn1b*-Tg mice [23]. Germ-free *Scnn1b*-Tg mice exhibit lung pathology, including morphological activation of macrophages, very similar to *Scnn1b*-Tg mice raised in specific pathogen free (SPF) conditions, indicating that the macrophages respond directly to the primary defect of airway surface dehydration and mucus stasis [13].

Genetic and pharmacologic studies suggest activation of multiple signaling pathways in response to defective mucus clearance in the *Scnn1b*-Tg mice. For example, genetic disruption of major pathways conventionally associated with airway inflammation and remodeling, e.g.*,* MyD88 and IL-4Rα, did not dramatically alter disease development [13, 24], highlighting the need to explore additional disease-promoting pathways. In the present study, we hypothesized that disease-associated molecular signatures linked to key host response, e.g.*,* airway inflammation and mucus cell metaplasia, could be identified by evaluating gene expression in selected tissues from *Scnn1b*-Tg mice at critical time points. Accordingly, we selected the following time points: 1) immediately after birth (PND 0, i.e., <24 hours), when the transgene is overexpressed but disease is not yet manifested histologically; 2) at PND 3, when tracheal mucus obstruction is prominent; 3) at PND 10, when chronic lower respiratory disease is being initiated; and 4) at PND 42, after establishment of chronic pulmonary disease. Gene-level and pathways analyses were used to generate a picture of differential gene expression in whole lung and macrophages. The results highlight a highly dynamic interplay of tissue-specific and time-dependent responses and set the stage for future studies to explore these complex interactions.

## Methods

### Mice and animal husbandry

Congenic C57BL/6N *Scnn1b*-Tg mice and WT littermates were maintained in a specific pathogen free (SPF) animal facility [10]. Germ-free (GF) mice were maintained in the National Gnotobiotic Rodent Resource Center at UNC [13]. Animals used in this study were maintained and studied under protocols approved by the University of North Carolina Institutional Animal Care and Use Committee.

### Lung RNA isolation

Tissues were collected from male mice at a designated time (1:00 pm). The lung left lobe was removed by cutting the extrapulmonary bronchus at the level of the hilum. Dissected tissue was stored in RNAzol (QIAzol lysis reagent, Qiagen Sciences, Valencia, CA) at -20°C. RNA was prepared using Qiagen RNeasy Mini Kit (Qiagen Sciences, Valencia, CA; following protocol recommended for animal tissue) followed by ammonium acetate precipitation. To minimize the effect of biological variation between individual animals, total RNA from three age- and genotype-matched mice were pooled to constitute each sample. A total of three RNA samples were analyzed at each time point for WT and *Scnn1b*-Tg mice.

### Macrophage RNA preparation

Male *Scnn1b*-Tg mice and WT littermates were anesthetized with an intraperitoneal administration of 2,2,2 tribromoethanol (T48402, Sigma, St. Louis, MO). The lungs were lavaged at least 4 times with calcium- and magnesium-free DPBS supplemented with 0.5 mM EDTA with the volume determined on weight-based formula [13]. Magnetic-activated cell sorting (MACS) was used to purify macrophages using Anti-Ly-6G MicroBead Kit (130-092-332, Miltenyi Biotech, MA). This approach selectively deplete granulocytes that predominantly express Ly-6G as a surface marker [25]. Since BAL cells include granulocytes, macrophages, lymphocytes and occasional dendritic and epithelial cells, the Ly-6G negative fraction, in addition to macrophages, is expected to include lymphocytes, dendritic cells and epithelial cells, but these cells are rare in these preparations. BALs cell pellets were suspended in 200 μl of MACS buffer. 50 μl of anti-Ly-6G-Biotin solution was added followed by incubation at 4°C for 10 min. Subsequently, 100 μl of anti-biotin microbeads and 150 μl of MACS buffer were added followed by incubation at 4°C for 15 minutes. Cell pellets washed with 10 ml MACS buffer were dissolved in 500 μl of MACS buffer. Thereafter, macrophage isolation through negative selection was carried out according to manufacturer’s recommendations. Ly-6G negative cells (predominantly macrophages) were pelleted, snap-frozen, and stored at -80°C. At PND 0, PND 3 and PND 10 time points, total macrophages collected from three genotype-matched pups were pooled to generate each sample. Each sample at PND 42 time point represents macrophages collected from individual mice. Frozen macrophage pellets were lysed and homogenized in lysis buffer and QIAshredder (Qiagen, Valencia, CA). Genomic DNA was eliminated using gDNA eliminator spin columns (Qiagen, Valencia, CA). The RNA was isolated using Purelink RNA mini kit (Invitrogen, NY).

### cDNA generation and microarray

12 ng RNA was used to generate cDNA using Ovation Pico WTA system V2 kit (NuGEN Technologies, CA). Total RNA or cDNA samples were submitted to the UNC Functional Genomics Core for cDNA preparation and hybridized to Affymetrix Mouse Gene 1.0 ST arrays according to the manufacturer’s instructions (Affymetrix Inc., Santa Clara, CA).

### Microarray data analysis

Probe level intensities from Affymetrix GeneChip Scanner 3000 in .CEL files were evaluated for quality by whole array statistics using Affymetrix Expression Console software. Gene expression analyses were performed using Partek Genomics Suite v6.6 (Partek Inc., St. Louis, MO). Briefly, probeset intensities were extracted from .CEL files by RMA background correction following GC content and sequence adjustments, normalized using quantile normalization, and gene level intensities were summarized through median polish, based on a modified meta-probeset mapping (.mps) derived from Affymetrix latest transcript annotation (release na33.2 mm9). The meta-probeset mapping consolidated all probesets to unique gene identifiers parsed from Affymetrix transcript annotation with the following precedence: ENSEMBL gene, Refseq mRNA, and Genbank nucleotide identifiers. Data quality, batch effect, and sample groupings were assessed by Principle Component Analysis (PCA) with correlation dispersion matrices. Differential gene expression was analyzed using ANOVA and linear contrast between experimental groups. The resultant differential expression (DE) p-values were adjusted for Multiple Test Correction using the False Discovery Rate (FDR) by Benjamini-Hochberg method [26].

Differentially expressed genes (DEG) were obtained through the combination of selected p-value and fold change filters (as indicated in figure legends), and normalized log2 intensities from individual arrays of DEGs were extracted for hierarchical clustering. Normalized log2 intensities were used in Gene Set Enrichment Analyses (GSEA) against gene sets derived from Biological Processes of the current Gene Ontology (GO) annotation database and custom gene sets relevant to lung disease and cellular physiology. Visual representations of GSEA enrichment FDR q-values from multiple sample groups were generated by hierarchical clustering of the transformed q-values using Cluster3 [27] and Java TreeView [28]. Detailed data mining of GSEA results from related GO vocabulary terms was performed by extracting relationships between functional terms from the current GO flat file download, and visualizing the resultant networks decorated by specific enrichment FDR q-values with Cytoscape [29]. The complete expression dataset has been submitted to the Gene Expression Omnibus (GEO) database with the accession number of GSE47551. http://www.ncbi.nlm.nih.gov/geo/query/acc.cgi?acc=GSE47551.

### Protein extraction and western blotting

Total protein was extracted from BAL macrophages after lysing with Radioimmuno Precipitation Assay buffer supplemented with 0.5 mM EDTA, 0.1 mM DDT and Halt protease inhibitors (Thermoscientific, Rockford, IL). Proteins were separated by SDS-PAGE (NuPage 4-12% Bis-Tris gradient gel) using MES buffer (Invitrogen, CA) and transferred to PVDF membranes. Rabbit antibodies against FIZZ-1 (39626, ABCAM), YM1/2 (a kind gift from Dr. Shioko Kimura, NCI, Maryland), and α-tubulin (T5168, Sigma) were used for the westerns. Protein bands were analyzed using Alexa fluor 680 Goat anti-rabbit IgG (A21109, Invitrogen) or IRdye800 anti-mouse IgG (610-132-121, Rockland). Quantification was performed using Odyssey and the data was normalized to α-tubulin. The data analysis was performed using unpaired two-tailed *t*-test on Graph-Pad Prism (La Jolla, CA).

## Results and discussion

### Whole lung gene expression patterns are altered by developmental age and *Scnn1b*-Tg expression

**Figure 1:**
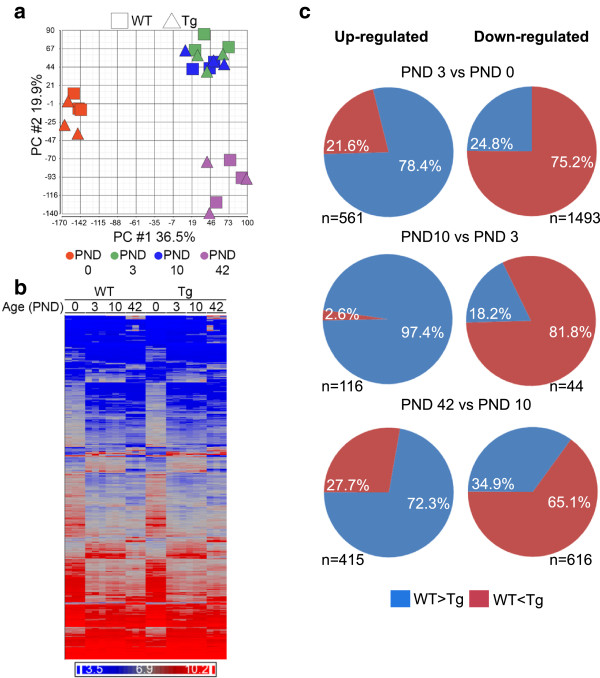
**Gene expression patterns in WT and** ***Scnn1b*** **-Tg whole lung. (a)** Principal component analysis (PCA) of gene expression from WT and *Scnn1b*-Tg whole lungs at PND 0, 3, 10, and 42 plotted in two-dimensional space using the first two principal components, which together constitute 55.4% of the overall variance in this study. Squares = WT; Triangles = *Scnn1b*-Tg. Each symbol represents the results of a single microarray. Each symbol represents a pool of animals as described in the methods. N = 3 pools for each age and genotype. Age is designated by color: PND 0 (red), PND 3 (green), PND 10 (blue), and PND 42 (purple). **(b)** Unsupervised hierarchical clustering of the combined set of differentially expressed genes (DEGs) that survive the filtering criteria (FDR ≤ 0.05, fold-change ≥ 2) across development (comparing PND 0 to all other time points for each genotype, i.e., WT and *Scnn1b*-Tg; total genes represented = 4514; Additional file 2: Results file S1). Dark blue indicates lower expression levels and bright red indicates higher expression levels, and each column represents the results of one microarray N = 3 for each genotype at each age). **(c)** Pie charts highlighting the shift in expression of developmentally regulated genes due to *Scnn1b*-Tg expression. Each row represents a different developmental interval and each pie chart represents the pattern for the genes that are differentially regulated (fold-change >2.0; FDR < 0.05) in WT mice. Genes normally up-regulated in WT mice are represented in the left column and genes normally down-regulated are represented in the right column, with the number of gene shown for each piechart. The percentages represent the genes that are either higher or lower (blue and red, respectively) in WT vs *Scnn1b*-Tg at the later developmental stage represented by the interval.

Principal Components Analysis (PCA) revealed that age was the primary factor affecting global gene expression in lung specimens (Figure 1a). PC1 (36.5% of over all variance) separated PND 0 mice from older animals, while PC2 (19.9% of overall variance) separated PND 3 and 10 from PND 42. *Scnn1b*-Tg mice clustered tightly with their WT littermates at all ages. By extending the analysis to evaluate PC4 and PC5, the WT mice began to separate from *Scnn1b*-Tg mice, but only at PND 10 and PND 42, respectively (Additional file 1: Figure S1a). This pattern was also observed upon hierarchical clustering of a combined set of 4514 differentially expressed genes (DEGs; FDR ≤ 0.05, FC ≥ 2) comparing PND 0 expression values to all other time points for each genotype (Figure 1b; Additional file 2: Results file S1). The heat-map demonstrates that a majority of changes in global gene expression (mainly down-regulation) occurred between PND 0 and PND 3, regardless of genotype.

As predicted from the PCA and the hierarchical clustering heat maps, robust differences in gene expression were present in both WT (Table 1) and *Scnn1b*-Tg (Table 2) mice across development, and the DEGs were qualitatively different among time intervals, consistent with the published reports highlighting the continual postnatal lung development [14] and gene expression profiling of developing murine lungs [30]. The top DEGs up-regulated from PND 0 to PND 3 in WT (Table 1) as well as *Scnn1b*-Tg (Table 2) mice were largely non-coding RNA species including long, non-coding RNAs and miRNAs. Non-coding RNAs tended to be robustly down-regulated between PND 3 to PND 10. While the specific functions of non-coding RNAs are only now being evaluated, their hypothesized role as master regulators of cell development controlling transcriptional regulatory circuitry is consistent with this finding [31, 32].

**Table 1:** **Developmentally regulated genes in whole lung from WT mice**

Gene Name	Fold change: PND 3 vs 0		Gene Name	Fold change: PND 10 vs 3		Gene Name	Fold change: PND 42 vs 10	
	WT	***Scnn1b*** -Tg		WT	***Scnn1b*** -Tg		WT	***Scnn1b*** -Tg
**UP-REGULATED**								
Vaultrc5*	28.5	27.7	*Clca3*	71.5	12.0	Snord116*	26.2	19.6
Gm22866*	24.4	26.8	*Chi3l4*	12.9	4.6	*Gm10722*	25.7	43.9
Snora16a*	20.3	22.0	*Chi3l3*	10.1	5.2	*Bpifa1*	23.0	7.3
Rnu3a*	18.4	16.6	*Crabp1*	8.9	5.6	*Inmt*	17.8	10.8
*Gm26493*	17.3	13.2	*Tff2*	8.0	7.9	*Cyp2b10*	17.6	10.1
Rnu3b1*	15.7	14.7	*Ltbp2*	6.8	3.9	*Gm10800*	17.6	67.7
*Gm23444*	13.8	13.3	*Hmcn1*	5.4	3.3	*Fmo3*	14.9	6.6
Snord22*	13.7	13.4	*C7*	5.4	7.4	*Lrat*	14.7	14.7
Snora69*	13.3	11.9	*Bpifb1*	4.2	3.1	*Cfd*	13.9	14.4
Rny1*	13.1	15.6	*Enpp1*	4.0	1.9	*Nr1d1*	13.7	14.4
n-R5s25*	12.4	13.7	*Muc5ac*	4.0	1.5	*C4b*	13.2	13.8
Snora23*	12.4	10.5	*Tnc*	3.9	3.0	Gm25089*	11.8	9.5
Snord118*	11.1	7.2	*A2m*	3.7	2.1	*Prelp*	11.6	11.6
*Gm24616*	10.9	8.4	*Muc5b*	3.7	2.0	*Serpina3n*	11.3	6.3
*Gm23927*	10.0	13.5	Mir27b*	3.6	1.7	Mir680-2*	11.2	9.0
**DOWN-REGULATED**								
Meg3*	-32.1	-34.3	*Gm26493*	-12.9	-16	*Tnc*	-25.1	-10
*C530030P08Rik*	-10.3	-19	Snora69*	-9.8	-9.6	*Egfem1*	-24	-18
*Zbtb16*	-8.8	-3.3	*Gm24616*	-8.8	-12.2	*Prss35*	-18.4	-9.2
Malat1*	-8.8	-9.7	Snord118*	-7.6	-4.7	*Agt*	-14.8	-9.3
6720401G13Rik*	-7.9	-11.7	Snora34*	-5.1	-6	*Vcan*	-14.4	-7.5
*Mir145*	-6.2	-11.9	*Olfm4*	-4.8	-3.9	*Spon2*	-12.7	-7.6
*Atp6v0a4*	-6.2	-2.2	*Asprv1*	-4.6	-1.8	*Slc27a6*	-12.7	-12.6
*Adamtsl2*	-6	-3	*Cldn4*	-4.5	-3.5	*Frem1*	-12.3	-6.4
*Wnk1*	-5.8	-9	*Stfa3*	-4.2	-3.4	*Clca3*	-11	11.6
*Dlk1*	-5.8	-4.1	H19*	-4.1	-1.5	6330403K07Rik*	-10.1	-4.8
*Tead1*	-5.7	-5.6	*Igf2*	-4	-3.1	*Ccna2*	-9.9	-7.8
*Nfat5*	-5.6	-7.6	*Agtr2*	-3.6	-3.2	*Crabp1*	-9.7	-4.8
*Cox20*	-5.5	-7.9	*S100a14*	-3.4	-3.9	*Stfa2l1*	-9.5	-8.7
*Tfcp2l1*	-5.3	-3.9	*Gdpd2*	-3.2	-2.6	*Phex*	-9.2	-5.5
*Leng8*	-5.2	-7.1	*Smpx*	-3.2	-1.3	*Chi3l4*	-8.9	10.5

Listing of the top 15 developmentally up- and down-regulated genes from whole lung of WT mice between three separate age intervals. The fold-changes for these top 15 genes are shown for both the WT and the *Scnn1b*-Tg mice.

* Non-coding RNA species.

**Table 2:** **Developmentally regulated genes in whole lung from** ***Scnn1b*** **-Tg mice**

Gene Name	Fold change: PND 3 vs 0		Gene Name	Fold change: PND 10 vs 3		Gene Name	Fold change: PND 42 vs 10	
	***Scnn1b*** -Tg	WT		***Scnn1b*** -Tg	WT		***Scnn1b*** -Tg	WT
**UP-REGULATED**								
Vaultrc5*	27.7	28.4	*Clca3*	12.0	71.4	*Gm10800*	67.8	17.6
Gm22866*	26.8	24.4	*Mmp12*	11.5	1.9	*Gm10722*	43.9	25.8
Snora16a*	22.0	20.3	*Tff2*	7.9	8.0	Snord116*	19.6	26.2
Rnu3a*	16.6	18.4	*C7*	7.4	5.4	*Lrat*	14.7	14.8
Rny1*	15.6	13.1	*Crabp1*	5.7	8.9	*Nr1d1*	14.4	13.8
Rnu3b1*	14.7	15.7	*Chi3l3*	5.2	10.1	*Cfd*	14.4	13.9
n-R5s25*	13.6	12.4	*Chi3l4*	4.6	13.0	*C4b*	13.8	13.2
Gm23927*	13.5	10.0	*H2-Aa*	4.6	2.6	*Chi3l3*	12.2	1.4
Snord22*	13.4	13.7	*H2-Ab1*	4.3	2.8	*Car3*	11.9	10.6
*Gm23444*	13.3	13.9	*Ltbp2*	3.9	6.8	*Prelp*	11.6	11.6
*Gm26493*	13.2	17.3	*4833424O15Rik*	3.8	2.7	*Clca3*	11.6	-10.9
Snora69*	11.9	13.3	*Cd74*	3.7	2.3	*Inmt*	10.8	17.8
Snora23*	10.5	12.4	*H2-Eb1*	3.7	2.6	*Chi3l4*	10.5	-8.9
Snord35b*	9.6	7.9	*Aard*	3.6	1.9	*Cxcl14*	10.2	8.0
Snora34*	9.3	9.6	*Cdkn2c*	3.4	3.3	*Speer8-ps1*	10.2	8.7
**DOWN-REGULATED**								
Meg3*	-34.3	-32.1	Gm26493*	-16.0	-12.3	*Egfem1*	-18.0	-24.0
*C530030P08Rik*	-19.0	-10.3	Gm24616*	-12.2	-8.8	H19*	-13.9	-5.5
Mir145*	-11.9	-6.2	Snora69*	-9.6	-9.8	*Slc27a6*	-12.6	-12.7
*6720401G13Rik*	-11.7	-7.9	Snora23*	-9.4	-5.5	*Tnc*	-10.0	-25.1
Malat1*	-9.7	-8.8	Snora34*	-6.0	-5.1	*Capn6*	-9.8	-7.6
*Gm7265*	-9.5	-4.5	*Gm10722*	-4.7	-1.7	*Agt*	-9.3	-14.8
*Wnk1*	-9.0	-5.8	*S100a14*	-3.9	-3.4	*Prss35*	-9.2	-18.4
Mir23b*	-8.6	-5.1	Gm22806*	-3.6	-3.1	*Pbk*	-8.8	-3.7
*Cox20*	-7.9	-5.5	Rnu1b1*	-3.6	-2.4	*Stfa2l1*	-8.7	-9.5
*Nfat5*	-7.6	-5.6	*Cldn4*	-3.5	-4.5	*Hist1h2ab*	-8.6	-7.3
Gm25831*	-7.2	-2.6	*Agtr2*	-3.2	-3.6	*Nuf2*	-8.3	-5.6
*Leng8*	-7.1	-5.2	*Igf2*	-3.1	-4.0	*Ccnb2*	-7.9	-5.6
*Fbxl7*	-5.9	-3.4	*Upk3a*	-3.1	-2.4	*Ccna2*	-7.8	-9.9
*B930095G15Rik*	-5.7	-3.7	*Cst8*	-2.8	-2.3	*Spon2*	-7.6	-12.7
*Srrm2*	-5.7	-4.2	*Liph*	-2.7	-1.9	*Vcan*	-7.5	-14.4

Top 15 developmentally up- and down-regulated genes from whole lung of *Scnn1b*-Tg mice between three separate age intervals. The fold-changes for these top 15 genes are shown for both *Scnn1b*-Tg and the WT mice.

* Non-coding RNA species.

Interestingly, many of the top up-regulated genes from PND 3 to PND 10 in both WT and *Scnn1b*-Tg mice are related to goblet cell (mucous cell) function and are frequently associated with Th2 inflammation in mice (*Clca3, Chi3l4, Chi3l3, Muc5ac, Muc5b, Tff2*) (Tables 1 and 2) [9, 33, 34]. This finding is consistent with the overall Th2 polarization characteristic of early postnatal immunity [35, 36] and parallels the wave of goblet cell appearance observed histologically during this time frame [10]. We speculate that these under-appreciated responses observed in developing lung are related to innate defense functions of mucus/mucus clearance during the early neonatal period. The fold-increase for this group of genes was always less in *Scnn1b*-Tg compared to WT during the PND 3–10 interval. Interestingly, while these genes were later down-regulated in WT mice during the PND 10–42 interval, they continued to be up-regulated further at the later interval in *Scnn1b*-Tg mice (Tables 1 and 2 and data not shown). Thus, failure to down-regulate these Th2 response-associated genes, e.g.*, Clca3, Chi3l3,* and *Chi3l4,* in the PND 10–42 interval is a key developmental shift that occurs as a consequence of *Scnn1b*-Tg expression. Also notable during the PND 3 to PND 10 interval was a large up-regulation of *Mmp12* in *Scnn1b*-Tg mice as compared to WT (Table 2), consistent with the role of *Mmp12* as a contributor to the airspace enlargement in this model [23].

While evaluation of individual gene-level differences was informative, interpretation of collective expression patterns was aided by Gene Set Enrichment Analysis (GSEA), which identified the top Gene Ontology groups that differed across developmental intervals (Table 3). GSEA analysis revealed that G-protein regulated signaling pathways capable of responding to various stimuli are established early in post-natal development (PND 3 versus PND 0) for both WT and *Scnn1b*-Tg mice (Table 3). Up- and down-regulated pathways were more similar between the two genotypes at the early (PND 3 versus PND 0) and late (PND 42 versus PND 10) intervals compared to the intermediate PND 10 versus PND 3 interval. The patterns observed in WT mice for the PND 3 to PND 10 interval suggest continued lung development based upon the up-regulation of pathways involved with reorganization of the extracellular matrix, epithelial cell migration, and continued maturation of vessels. During the same interval, down-regulation of pathways involved in defense and killing of pathogens suggests the establishment of immune homeostasis in WT mice. Significant development of an adaptive immune response signature occurred during the PND 10 and PND 42 interval in both lines of mice as indicated by up-regulation of GO pathways involving humoral immune responses, lymphocyte immunity, antigen processing, and complement activation.

**Table 3:** **Developmentally regulated Gene Ontology groups for whole lung in WT and** ***Scnn1b-*** **Tg mice**

PND 3 versus PND 0		PND 10 versus PND3		PND 42 versus PND 10	
WT	***Scnn1b*** -Tg	WT	***Scnn1b*** -Tg	WT	***Scnn1b*** -Tg
**UP-REGULATED**					
***GO:0007608***	***GO:0007608***	GO:0030198	GO:0007067	***GO:0006959***	***GO:0006959***
***Sensory perception of smell***	***Sensory perception of smell***	Extracellular matrix organization	Mitosis	***Humoral immune response***	***Humoral immune response***
***GO:0007606***	***GO:0007606***	GO:0043062	GO:0000280	***GO:0048002***	***GO:0019882***
***Sensory perception of chemical stimulus***	***Sensory perception of chemical stimulus***	Extracellular structure organization	Nuclear division	***Antigen processing and presentation of peptide antigen***	***Antigen processing and presentation***
***GO:0007600***	***GO:0007600***	GO:0021988	GO:0007059	***GO:0002455***	GO:0002253
***Sensory perception***	***Sensory perception***	Olfactory lobe development	Chromosome segregation	***Humoral immune response mediated by circulating immunoglobulin***	Activation of immune response
***GO:0019236***	***GO:0019236***	GO:0030199	GO:0048285	***GO:0072376***	GO:0050778
***Response to pheromone***	***Response to pheromone***	Collagen fibril organization	Organelle fission	***Protein activation cascade***	Positive regulation of immune response
GO:0009263	***GO:0007186***	GO:0031589	GO:0051301	GO:0006956	***GO:0048002***
Deoxyribonucleotide biosynthetic process	***G-protein coupled receptor signaling pathway***	Cell-substrate adhesion	Cell division	Complement activation	***Antigen processing and presentation of peptide antigen***
***GO:0007186***	GO:0002861	GO:0071526	GO:0000278	GO:0002474	***GO:0072376***
***G-protein coupled receptor signaling pathway***	Regulation of inflammatory response to antigenic stimulus	Semaphorin-plexin signaling pathway	Mitotic cell cycle	Antigen processing and presentation of peptide antigen via MCH class I	***Protein activation cascade***
GO:0033108	GO:0002675	GO:0007155	GO:0007051	***GO:0019882***	***GO:0006956***
Mitochondrial respiratory chain complex assembly	Positive regulation of acute inflammatory response	Cell adhesion	Spindle organization	***Antigen processing and presentation***	***Complement activation***
GO:0009262	GO:0050877	GO:0002040	GO:0031023	GO:0006958	***GO:0002455***
Deoxyribonucleotide metabolic process	Neurological system process	Sprouting angiogenesis	Microtubule organizing center organization	Complement activation classical pathway	***Humoral immune response mediated by circulating immunoglobulin***
GO:0006270	GO:0050715	GO:0090132	GO:0007052	GO:0002449	GO:0002684
DNA replication initiation	Positive regulation of cytokine secretion	Epithelial migration	Mitotic spindle organization	Lymphocyte mediated immunity	Positive regulation of immune system process
GO:0032981	GO:0046146	GO:0021772	GO:0051297	GO:0017144	GO:0002478
Mitochondrial respiratory chain complex I assembly	Tetrahydrobiopterin metabolic process	Olfactory bulb development	Centrosome organization	Drug metabolic processes	Antigen processing and presentation of exogenous peptide antigen
**DOWN-REGULATED**					
***GO:0007265***	***GO:0051056***	GO:0044364	GO:0008299	***GO:0007067***	***GO:0007067***
***Ras protein signal transduction***	***Regulation of small GTPase mediated signal transduction***	Disruption of cells of other organism	Isoprenoid biosynthetic process	***Mitosis***	***Mitosis***
***GO:0051056***	***GO:0007265***	GO:0031640	NONE*	***GO:0000280***	***GO:0000280***
***Regulation of small GTPase mediated signal transduction***	***Ras protein signal transduction***	Killing of cells of other organism		***Nuclear division***	***Nuclear division***
***GO:0046578***	***GO:0046578***	GO:0051818		***GO:0048285***	***GO:0048285***
***Regulation of Ras protein signal transduction***	***Regulation of Ras protein signal transduction***	Disruption of cells of other organism involved in symbiotic interaction		***Organelle fission***	***Organelle fission***
***GO:0007266***	***GO:0007266***	GO:0051883		***GO:0007059***	***GO:0007059***
***Rho protein signal transduction***	***Rho protein signal transduction***	Killing of cells in other organism involved in symbiotic interaction		***Chromosome segregation***	***Chromosome segregation***
GO:0035295	GO:0016568	GO:0006953		***GO:0051301***	***GO:0051301***
Tube development	Chromatin modification	Acute phase response		***Cell division***	***Cell division***
GO:0010631	GO:0016569	GO:0050829		***GO:0006323***	***GO:0000278***
Epithelial cell migration	Covalent chromatin modification	Defense response to Gram-negative bacterium		***DNA packaging***	***Mitotic cell cycle***
GO:0090130	GO:0016570	GO:0002886		***GO:0000278***	***GO:0006323***
Tissue migration	Histone modification	Regulation of myeloid leukocyte mediated immunity		***Mitotic cell cycle***	***DNA packaging***
GO:0090132	GO:0007507	GO:0051873		GO:0000819	***GO:0071103***
Epithelium migration	Heart development	Killing by host of symbiont cells		Sister chromatid segregation	***DNA conformation change***
GO:0060562	GO:0046777	GO:0051852		GO:0000070	GO:0022402
Epithelial tube morphogenesis	Protein autophosphorylation	Disruption by host of symbiont cells		Mitotic sister chromatid segregation	Cell cycle process
GO:0035239	GO:0072358	GO:0031424		***GO:0071103***	GO:0034470
Tube morphogenesis	Cardiovascular system development	Keratinization		***DNA conformation change***	ncRNA processing

Top ten developmentally up- and down-regulated Gene Ontology groups for whole lung in WT and *Scnn1b-*Tg mice between the specified developmental intervals. Gene Ontology groups in common between the WT and *Scnn1b*-Tg line are highlighted by bolded and italicized text. Groups are only listed if FDR <0.1.

*NONE indicates that no additional groups met the significance threshold FDR<0.1.

*Scnn1b*-Tg mice showed early evidence of inflammatory signaling during the PND 0 to PND 3 interval as indicated by up-regulation of GO inflammatory response and cytokine signaling pathways (Table 3). Up-regulated GO groups related to mitosis at the PND3 to PND 10 interval in *Scnn1b*-Tg mice point to transgene expression-induced shifts in the mitotic state of the lung. Furthermore, the down-regulation of GO immune defense pathways seen in WT mice during the PND 3 to PND 10 interval was clearly disrupted in the *Scnn1b*-Tg line. Indeed, closer evaluation identified a pattern whereby expression of the transgene altered normal developmental processes in subtle, but measureable, ways that were not immediately apparent (Figure 1c). The absolute expression level of developmentally up-regulated genes was consistently less in the *Scnn1b*-Tg mice compared to WT, and at PND 10, a striking 97% of these genes had lower expression in *Scnn1b*-Tg mice compared to WT (Figure 1c). An inverse phenomenon held true for genes that were developmentally down-regulated in WT mice, which trended towards higher expression in *Scnn1b*-Tg mice. While the fold-changes between *Scnn1b*-Tg and WT for these genes were generally subtle, the consistency of the pattern leads to the conclusion that expression of the transgene, and/or its resultant pathology, alters developmental pathways. The presence of an inflammatory stimulus in the context of developing lung tissue during this early post-natal timeframe in this model is highly relevant to human bronchopulmonary dysplasia (BPD), whereby the under-developed lungs of premature infants are subjected to inflammatory challenges leading to long-term consequences for lung health [37].

**Figure 2:**
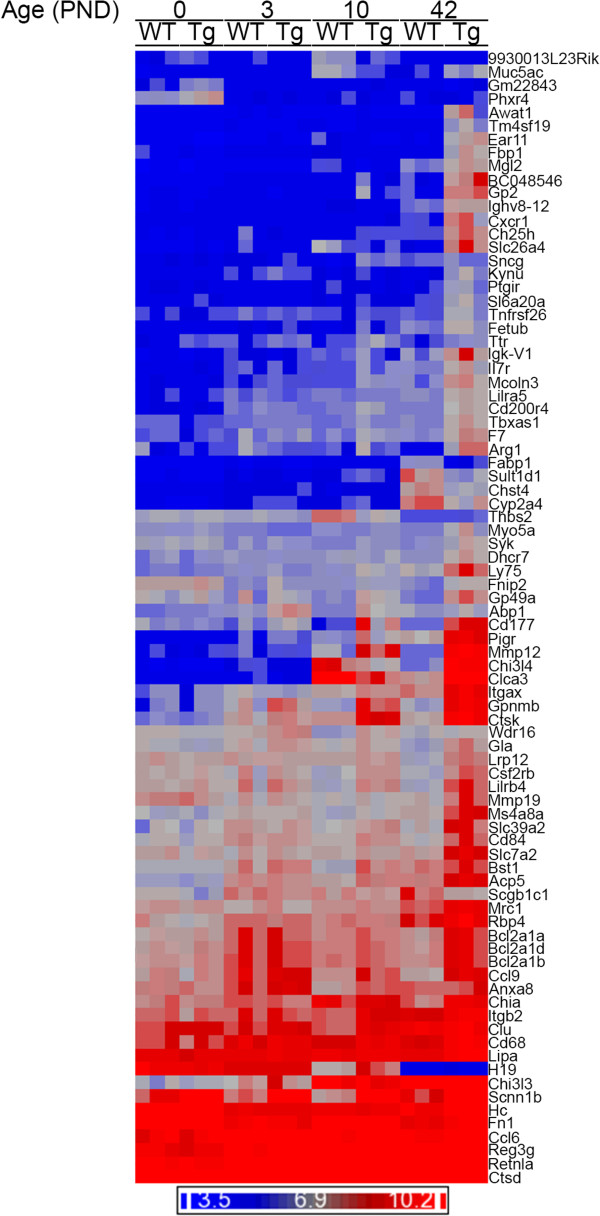
**Differential expression of genes in** ***Scnn1b*** **-Tg lungs compared to WT.** Unsupervised hierarchical clustering of the combined set of DEGs that survive the filtering criteria (FDR ≤ 0.05, fold-change ≥2) for WT versus *Scnn1b*-Tg for any time point; total genes represented = 84 (Additional file 2: Results file S1). Dark blue indicates lower expression levels and bright red indicates higher expression levels and each column represents the results of one microarray (n = 3 pools for each time point and genotype). DEGs appearing more than once reflect alternative probeset annotations on the Affymetrix microarrays. *Scnn1b* and *Ttr* differential gene expression reflects the overexpression from the transgenic construct used to generate the *Scnn1b*-Tg mice [9].

We next conducted analyses whereby the specific genes and pathways altered by transgene expression at the four time points were determined (Figure 2; Tables 3 and 4; Additional file 3: Table S1). Despite the robust pathological findings consistently observed in *Scnn1b*-Tg mice after PND 3 (neutrophilia, macrophage activation, airspace enlargement, mucus plugging [9, 12]), overexpression of the *Scnn1b* transgene in the club cells produced surprisingly few significant (FDR ≤ 0.05, FC ≥ 2) gene expression changes as evaluated from whole lung RNA (Additional file 2: Results file S1): only 84 combined DEGs (3, 2, 16, and 72, at PND 0, 3, 10, and 42, respectively), were identified between *Scnn1b*-Tg and WT mice (Figure 2; Table 4). As expected, the *Scnn1b* transgene was up-regulated at all time-points (Table 4). A majority of DEGs were up-regulated only at PND 42, with only a few genes (for example: *Scgb1c1, Cyp2a4, Fabp1*) robustly down-regulated, and very few genes differentially expressed at PND 0 and PND 3 time points. The down-regulated group at PND 10 included genes (*Muc5ac, Clca3, Slc26a4, and Chi3l4)*, associated with Th2 inflammatory processes and mucous cell functions, as described above (Tables 1 and Table 2).

**Table 4:** **Differentially expressed genes between WT and** ***Scnn1b*** **-Tg whole lungs**

Gene Name	PND 0		PND 3		PND 10		PND 42	
	Fold- Change	FDR ***p-value***	Fold-Change	FDR ***p-value***	Fold-Change	FDR ***p-*** value	Fold-Change	FDR ***p*** -value
*Scnn1b*	**11.7**	*8.47E-05*	**14.8**	*2.23E-05*	**14.0**	*3.10E-05*	**10.4**	*2.38E-05*
*Ttr*	1.5		1.4		**2.2**	*1.98E-02*	1.8	
*9930013L23Rik*	1.2		-1.2		*-2.2*	*4.20E-02*	1.2	
*Thbs2*	-1.0		-1.1		*-2.6*	*6.25E-05*	1.1	
*Fabp1*	-1.0		-1.1		-1.1		*-2.1*	*3.69E-04*
*Chst4*	1.1		1.0		1.1		*-2.1*	*1.35E-04*
*Scgb1c1*	-1.5		-1.2		-1.2		*-2.3*	*4.87E-02*
*Cyp2a4*	-1.0		1.1		1.1		*-2.4*	*1.91E-02*
*Sult1d1*	-1.1		-1.1		1.1		*-2.8*	*9.46E-03*
*Chi3l4*	-1.1		-1.1		*-3.1*	*5.78E-02*	**30.1**	*5.77E-07*
*Clca3*	-1.0		1.0		*-5.8*	*6.25E-05*	**21.9**	*4.37E-08*
*Slc26a4*	-1.1		-1.2		*-2.0*	*4.26E-01*	**10.3**	*7.47E-05*
*Muc5ac*	1.0		1.1		*-2.6*	*1.23E-02*	**2.6**	*2.34E-03*
*Phxr4*	**2.2**	*3.64E-02*	-1.0		-1.1		1.0	
*Gm22843*	**2.1**	*2.67E-02*	1.0		1.0		-1.0	
*Abp1 (Aoc1)*	1.2		**2.8**	*2.41E-02*	**2.3**	1.66E-02	1.1	
*Gpnmb*	1.1		**2.1**	*7.69E-01*	**5.5**	5.45E-02	**13.4**	*7.53E-04*
*Bst1*	-1.0		1.4		**2.8**	2.02E-02	1.6	
*Sncg*	1.1		-1.0		**2.4**	*1.39E-02*	1.2	
*Wdr16*	-1.2		1.6		**2.1**	*1.23E-02*	1.2	
*Anxa8*	-1.1		1.3		**2.1**	1.41E-02	1.1	
*Clu*	1.1		1.4		**2.1**	1.40E-02	1.1	
H19*	-1.3		-1.0		**2.6**	1.46E-02	1.0	
*Mmp12*	-1.0		-1.1		**5.4**	*4.53E-03*	**34.8**	*2.96E-07*
*Cd177*	-1.2		1.8		**6.6**	*2.69E-02*	**11.3**	*1.52E-03*
*Ctsk*	1.0		1.4		**2.7**	*1.41E-02*	**9.5**	*1.19E-06*
*Pigr*	1.1		1.5		**2.3**	*2.92E-01*	**4.9**	*3.40E-03*
*Gp49a*	1.1		1.2		**2.3**	*4.63E-01*	**4.8**	*1.94E-02*
*Lilrb4*	1.1		1.3		**2.4**	*3.56E-01*	**4.2**	*1.82E-02*
*Ccl9*	-1.2		1.0		**2.0**	*5.04E-01*	**3.9**	*1.79E-02*
*Mcoln3*	-1.0		1.1		**2.0**	*1.67E-01*	**3.4**	*2.13E-03*
*Cd68*	-1.1		1.3		**2.1**	*1.14E-01*	**2.8**	*6.81E-03*
*Gp2*	-1.0		-1.0		1.8		**9.9**	*2.63E-04*
*Arg1*	-1.2		-1.3		1.7		**8.9**	*6.84E-03*
*BC048546*	-1.1		1.1		1.8		**7.5**	*7.47E-05*
*Ear11*	-1.1		1.0		-1.5		**7.2**	*4.73E-06*
*Awat1*	1.0		1.0		1.0		**6.6**	*3.59E-03*
*Ch25h*	-1.0		-1.2		1.4		**6.2**	*1.30E-04*
*Chi3l3*	1.1		1.3		-1.5		**5.8**	*9.46E-03*
*Cxcr1*	1.0		-1.0		1.2		**5.7**	*9.66E-04*
*Bcl2a1d*	-1.2		-1.1		-1.3		**5.4**	*1.91E-02*
*Retnla (Fizz1)*	-1.4		-1.2		-1.9		**5.0**	*4.84E-02*
*Itgax*	-1.1		-1.3		1.6		**4.4**	*4.59E-03*
*Igk-V1*	-1.0		-1.2		1.0		**3.9**	*2.64E-02*
*Fbp1*	-1.2		-1.1		-1.4		**3.8**	*8.44E-05*
*Chia*	-1.1		-1.2		-1.3		**3.5**	*9.69E-03*
*Ccl6*	-1.0		1.3		1.2		**3.5**	*4.13E-02*
*Ly75*	1.0		-1.1		1.6		**3.3**	*7.53E-04*
*Ighv8-12*	1.1		-1.1		-1.1		**3.2**	*1.62E-06*
*Tm4sf19*	1.0		1.0		1.1		**3.2**	*3.91E-05*
*Slc39a2*	1.1		-1.1		1.7		**3.2**	*3.42E-02*
*Kynu*	1.0		1.2		1.1		**3.0**	*5.52E-03*
*Ms4a8a*	-1.0		-1.0		1.3		**3.0**	*4.18E-04*
*Reg3g*	-1.3		1.7		1.7		**3.0**	*4.08E-02*
*Csf2rb*	-1.1		1.3		1.9		**3.0**	*2.08E-02*
*Mgl2*	-1.0		-1.0		-1.6		**2.9**	*4.19E-04*
*Slc7a2*	-1.1		1.2		1.1		**2.9**	*9.46E-03*
*Cd84*	-1.1		-1.0		1.4		**2.9**	*1.53E-03*
*F7*	-1.2		1.3		1.7		**2.9**	*1.94E-02*
*Gla*	-1.2		1.4		1.3		**2.7**	*7.90E-03*
*Itgb2*	-1.3		1.1		1.7		**2.7**	*1.66E-02*
*Mmp19*	-1.0		1.3		1.1		**2.6**	*3.25E-02*
*Tbxas1*	-1.3		1.1		1.2		**2.6**	*2.76E-03*
*Lrp12*	-1.1		-1.1		1.2		**2.6**	*2.38E-05*
*Bcl2a1a*	1.1		-1.1		1.2		**2.6**	*1.27E-02*
*Slc6a20a*	1.1		-1.0		1.2		**2.5**	*6.17E-03*
*Cd200r4*	-1.1		-1.0		1.5		**2.5**	*1.52E-03*
*Rbp4*	-1.4		-1.1		-1.0		**2.4**	*1.94E-02*
*Lipa*	-1.3		1.1		1.1		**2.4**	*5.62E-04*
*Bcl2a1b*	1.1		-1.0		1.2		**2.4**	*2.06E-02*
*Il7r*	-1.0		1.0		1.5		**2.4**	*3.71E-02*
*Fn1*	-1.0		-1.2		-1.4		**2.3**	*4.24E-02*
*Mrc1*	-1.0		1.1		1.1		**2.3**	*1.14E-02*
*Ctsd*	-1.1		1.2		1.4		**2.3**	*4.87E-04*
*Hc*	1.1		-1.1		-1.3		**2.1**	*1.94E-02*
*Ptgir*	-1.0		1.1		-1.1		**2.1**	*1.91E-02*
*Lilra5*	1.0		-1.2		1.1		**2.1**	*3.40E-03*
*Dhcr7*	-1.1		-1.1		1.1		**2.1**	*2.55E-02*
*Tnfrsf26*	-1.0		1.0		1.2		**2.1**	*1.29E-02*
*Fnip2*	1.1		1.4		1.2		**2.1**	*1.33E-02*
*Acp5*	-1.0		1.0		1.7		**2.1**	*9.46E-03*
*Myo5a*	1.1		-1.2		1.0		**2.0**	*2.85E-02*
*Syk*	-1.0		1.1		1.2		**2.0**	*1.68E-02*
*Fetub*	-1.1		1.3		1.5		**2.0**	*3.92E-02*

Differentially expressed (fold-change >2; FDR<0.05) genes between WT versus *Scnn1b*-Tg from whole lung. Genes are listed if they were significant at one or more of the four developmental stages measured. Fold-change represents *Scnn1b*-Tg:WT. Up-regulated and down-regulated fold-changes are highlighted bold and *italics*, respectively.

Since *Scnn1b*-Tg expression is driven by the promoter for *Scgb1a1* gene (encoding club cell secretory protein), and because club cells are transiently necrotic during early postnatal life [9], we looked specifically at *Scgb1a1* gene expression as a surrogate for club cell function. Levels of *Scgb1a1* were modestly reduced in *Scnn1b*-tg mice compared to WT at PND 0, 3 and 10 [fold-change -1.9 (p-value 0.009), -1.7 (p-value 0.02), -1.5 (p-value 0.06), respectively], but not at PND 42 [fold-change -1.04 (p-value 0.82)]. These data indicate that normal club cell function was disrupted during early post-natal life as a result of transgene expression, as expected from histological findings, and that disruption of club cell function may be contributing to early inflammatory processes [38]. However, in the chronic state of muco-obstruction observed at PND 42, club cell function as measured by *Scgb1a1* expression was normal.

Evaluation of Gene Ontology terms associated with the differentially regulated genes was only moderately informative, since the genes belonged to multiple annotation categories that only rarely overlapped (Additional file 3: Table S1). However, after a review of the literature, most of the DEGs at PND 42, i.e., when chronic disease is firmly established, fell into expected functional categories, e.g., genes broadly related to lung inflammatory processes (*Itgb2*, *Kynu*, *Ptgir*), neutrophil influx (*Bst1*, *Cd177*, *Cxcr1*), activation of adaptive immunity (*Cd84*, *Ch25h*, *Gla*, *Il7r*, *Mmp19*), dendritic cells (*Itgax, Ly75*); macrophages (*Bcl2a1, Ccl6, Ccl9, Cd68*, *Ctsk*, *Ch25h*, *Gpnmb*, *Hc, Lilrb4*) or epithelial responses to stimuli (*Clca3*, *Ctsd*, *Gp2*, *Muc5ac*, *Pigr*, *Slc26a4*). A significant number of the DEGs were associated with classic Th2 inflammatory responses (*Ccl6*, *Chia*, *Chi3l4*, *Clca3*, *Ear11*, *F7*, *Itgax*, *Slc26a4*, *Tbxas1*) and alternative (M2) macrophage polarization (*Retnla, Arg1*, *Chi3l3*, *Chi3l4*, *Ch25h*, *Lipa*, *Mgl2*, *Mmp12*, *Mrc1*, *Tbxas1*) (Table 4 and Additional file 3: Table S1).

Analyses of GSEA pathways differentially expressed between WT and *Scnn1b*-Tg mice at the different time points confirmed up-regulation of inflammatory responses starting at PND 10 (Additional file 1: Figure S2; Additional file 4: Results file S2; Additional file 5: Results file S3) and revealed novel responses associated with the establishment of obstructive lung disease, such as up-regulation of GO cilia-specific pathways at PND 10, alterations in tissue organization and development (pathways up-regulated at PND 0 and down-regulated at PND 3) and possible disturbances in the establishment of lung immune homeostasis (pathways down-regulated at PND 0) (Table 5). By PND 42, there were no down-regulated pathways that met the significance threshold.

**Table 5:** **Differentially regulated Gene Ontology groups from whole lung between WT and** ***Scnn1b-*** **Tg mice**

PND 0	PND 3	PND 10	PND 42
**UP-REGULATED**			
GO:0031424	NONE*	GO:0006953	GO:0006954
Keratinization		Acute-phase response	Inflammatory response
GO:0035195		GO:0002526	GO:0050715
Gene silencing by miRNA		Acute inflammatory response	Positive regulation of cytokine secretion
GO:0090505		GO:0050707	GO:0001816
Epiboly involved in wound healing		Regulation of cytokine secretion	Cytokine production
GO:0090504		GO:0003341	GO:0050663
Epiboly		Cilium movement	Cytokine secretion
GO:0035194		GO:0050715	GO:0002444
Posttranscriptional gene silencing by RNA		Positive regulation of cytokine secretion	Myeloid leukocyte mediated immunity
GO:0044319		GO:0006954	GO:0002274
Wound healing, spreading of cells		Inflammatory response	Myeloid leukocyte activation
GO:0035278		GO:0045087	GO:0050707
Negative regulation of translation involved in gene silencing by miRNA		Innate immune response	Regulation of cytokine secretion
GO:0045974		GO:0032640	GO:0043299
Regulation of translation, ncRNA-mediated		Tumor necrosis factor production	Leukocyte degranulation
GO:0035313		GO:0002886	GO:0006955
Wound healing, spreading of epidermal cells		Regulation of myeloid leukocyte mediated immunity	Immune response
GO:0040033		GO:0044782	GO:0050729
Negative regulation of translation, ncRNA-mediated		Cilium organization	Positive regulation of inflammatory response
**DOWN-REGULATED**			
GO:0048002	GO:0007059	GO:0043931	NONE*
Antigen processing and presentation of peptide antigen	Chromosome segregation	Ossification involved in bone maturation	
GO:0009410	GO:0007067	GO:0061298	
Response to xenobiotic stimulus	Mitosis	Retina vasculature development in camera-type eye	
GO:0006805	GO:0000280	GO:0060039	
Xenobiotic metabolic process	Nuclear division	Pericardium development	
GO:0002495	GO:0048285	GO:0070977	
Antigen processing and presentation of peptide antigen via MHC class II	Organelle fission	Bone maturation	
GO:0071466	GO:0051301	GO:0002067	
Cellular response to xenobiotic stimulus	Cell division	Glandular epithelial cell differentiation	
GO:0002367	GO:0071103	GO:0030198	
Cytokine production involved in immune response	DNA conformation change	Extracellular matrix organization	
GO:0002374	GO:0006260	GO:0043062	
Cytokine secretion involved in immune response	DNA replication	Extracellular structure organization	
GO:0002478	GO:0006261	GO:0097435	
Antigen processing and presentation of exogenous peptide antigen	DNA-dependent DNA replication	Fibril organization	
GO:0034381	GO:0051297	GO:0007044	
Plasma lipoprotein particle clearance	Centrosome organization	Cell-substrate junction assembly	
GO:0097006	GO:0000278	GO:0050919	
Regulation of plasma lipoprotein particle levels	Mitotic cell cycle	Negative chemotaxis	

Top ten differentially up- and down-regulated Gene Ontology groups from whole lung between WT and *Scnn1b-*Tg mice at the four developmental stages. Groups are only listed if FDR <0.1.

NONE indicates that no groups reached the significance threshold (FDR<0.1).

Overall, these data suggest that defective airway mucus clearance due to *Scnn1b*-Tg overexpression, although originated in a relatively small compartment, i.e.*,* the airway epithelia, which comprises less than 2% of the total lung surface area, can lead to transcriptional modifications that affect other lung compartments and cell populations, e.g.*,* parenchymal and myeloid lineages, which are strong enough to be detected in whole lung preparations. However, the relative dearth of robust gene-level signatures, especially at the earlier time points, suggested that evaluating gene expression in purified cell populations would be informative. Due to the robust morphological activation of macrophages in the *Scnn1b*-Tg mice, the tendency for genes involved in macrophage function to be up-regulated in whole lung (Additional file 3: Table S1), and the importance of this cell type in lung disease pathogenesis, we continued our studies by evaluating gene expression in purified pulmonary macrophages.

### Macrophage DEGs between *Scnn1b*-Tg and WT are robust and dynamic

We hypothesized that defective mucus clearance would alter lung macrophage gene expression and, consequently, we studied purified BAL macrophages from WT and *Scnn1b*-Tg mice at the previously utilized developmental stages. Furthermore, to evaluate the contribution of lung bacterial infections, the gene expression profiles of lung macrophages purified from germ-free (GF) *Scnn1b*-Tg and WT littermates at PND 42 were also studied. In addition to macrophages, the harvested BAL preparations includes lymphocytes, eosinophils, and neutrophils, with the contribution of each cell type varying between WT and *Scnn1b*-Tg and among developmental time points. To minimize granulocytes proportions in the BAL, macrophages were purified by negative selection for Ly6G expression (a marker exclusively expressed on neutrophils and eosinophils), and pools with 95.86% ± 0.25% (SEM) purity were obtained (Additional file 1: Figure S3 and Additional file 3: Table S2). Lack of Ly-6G expression in all macrophage preparations used in this study was confirmed by gene array data (data not shown).

**Figure 3:**
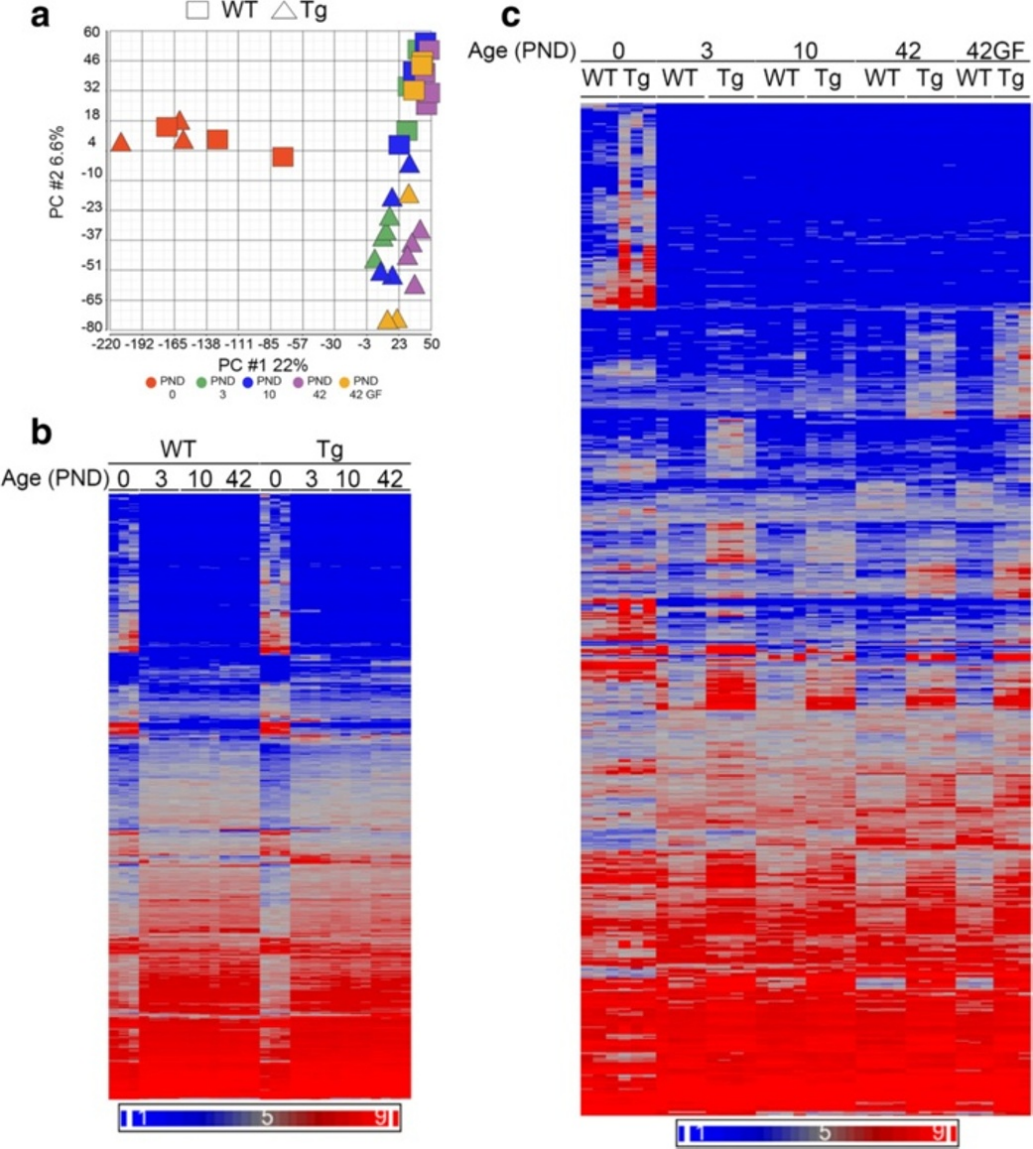
**Gene expression patterns in purified BAL macrophages. (a)** PCA plot as in Figure 1a for purified macrophages from WT (Squares) and *Scnn1b*-Tg (Triangles) mice at PNDs 0 (red), 3 (green), 10 (blue), 42 (purple) and for germ-free (GF) macrophages at PND 42 (orange). PC#1 (22% of the overall variance) separates the PND 0 from other ages; PC#2 (6.6% of the overall variance) separates WT from *Scnn1b*-Tg. **(b)** Unsupervised hierarchical clustering of the combined set of developmentally regulated DEGs as determined for Figure 1b except from purified macrophages (total number of genes represented = 4763; Additional file 2: Results file S1). **(c)** Unsupervised hierarchical clustering of the combined set of DEGs in macrophages filtered as in Figure 2 for WT versus *Scnn1b*-Tg (total genes represented = 1320; Additional file 2: Results file S1). For this figure, symbols, color coding and filtering were as in Figure 1. GF = germ-free.

PCA analysis of purified macrophage arrays showed both age and genotype as drivers of global gene expression variation (Figure 3a). PC1 separated macrophages at PND 0 from later time points. However, PC2 separated macrophages from *Scnn1b*-Tg and WT mice at PND 3, PND 10, and PND 42, indicating disease-specific activation of gene signatures. PND 0 WT and *Scnn1b*-Tg macrophages separated primarily in PC5, with other PCs reflecting either age or other unexplained variation (Additional file 1: Figure S1b). Interestingly, macrophages purified from germ-free (GF) mice clustered close to their age-matched SPF counterparts.

As with the whole lung, the population of macrophages purified from the BAL exhibited robust developmental patterns, evident at all time points (Tables 6, 7 and 8). Evaluation of the top-signaling genes generated a complex picture with a number of obvious differences between WT and *Scnn1b*-Tg mice, especially noticeable at the PND 42 versus PND 10 interval, where genes down-regulated in WT mice were up-regulated in *Scnn1b*-Tg mice (Table 6) and vice versa (Table 7). The especially robust differential gene expression between the PND 0 and PND 3 time point (Figure 3b; Additional file 2: Results file S1) is consistent with previous studies identifying this interval as a key interval for alveolar macrophage differentiation [39]. Robust up-regulation of *Siglec5* and *Itgax* (also known as *SiglecF* and *Cd11c*; fold-change 7.7 and 5.0, and FDR 1.0E-10 and 2.7E-9, respectively) between PND 0 and PND 3, which then stabilized between all other intervals (fold changes < 1.5; FDR >0.3; not shown), confirmed the previous observations that these cell surface markers appear suddenly and that they define the resident alveolar macrophage population immediately after birth in mice [39]. This dataset may be especially useful to identify transcriptional changes that accompany *Siglec5* and *Itgax* up-regulation during this critical time.

**Table 6:** **Developmentally regulated genes in purified macrophages from WT mice**

Gene Name	Fold-change: PND 3 vs 0		Gene Name	Fold-change: PND 10 vs 3		Gene Name	Fold-change: PND 42 vs 10	
	WT	***Scnn1b*** -Tg		WT	***Scnn1b*** -Tg		WT	***Scnn1b*** -Tg
**UP-REGULATED**								
*Fabp1*	42.2	8.6	*Ear11*	39.5	10.5	*Spag11b*	23.8	21.0
*Coro6*	24.3	3.0	*Fbp1*	18.0	3.8	*Cpne5*	18.7	7.0
*Treml4*	24.1	13.2	*Ccl24*	12.2	4.6	*Slc9a2*	10.2	2.5
*Rnase6*	14.1	6.0	*Retnlg*	12.0	-2.1	*Lrg1*	7.4	3.1
F630028O10Rik*	13.5	12.3	*Retnla*	9.8	1.0	*Pnpla5*	6.5	2.0
*Gm4070*	10.8	4.7	*Ccl17*	9.4	5.0	*Ucp3*	6.4	5.8
*AW112010*	10.6	13.9	*Alox15*	4.3	1.9	*Epcam*	6.2	1.5
*Mcoln3*	9.4	18.6	*Arg1*	4.1	-1.8	*Gca*	5.4	3.1
*Gbgt1*	8.6	8.3	*Prg2*	3.6	1.4	*5730507C01Rik*	5.4	3.5
*Irg1*	8.5	118.2	*Ch25h*	3.3	3.9	*D630039A03Rik*	5.3	6.2
*Alox5*	8.5	4.1	*H2-Ab1*	3.1	2.2	Gm26154*	4.8	1.5
*2010016I18Rik*	8.4	6.5	*H2-Aa*	3.0	2.0	*Cd74*	4.8	5.9
*Rab44*	8.3	3.1	*Il13*	3.0	1.4	*Gal*	4.8	2.1
*Cfb*	8.3	11.4	*Mmp12*	3.0	1.9	*H2-Ab1*	4.7	5.8
*Fpr1*	8.0	15.5	*Serpine1*	2.9	1.6	*Tnfsf13b*	4.6	-1.1
**DOWN-REGULATED**								
*Agr2*	-223.2	-171.4	*Hp*	-10.4	-5.4	*Ear11*	-44.3	14.0
*Chad*	-105.2	-163.4	*Scgb1a1*	-9.7	-4.1	*Fbp1*	-19.1	15.8
*Muc5b*	-83.9	-113.5	*Saa3*	-8.6	-51.9	*S100a9*	-16.8	-3.0
Meg3*	-65.4	-84.1	*Irg1*	-7.8	-6.3	*Arg1*	-14.3	2.4
*Lypd2*	-64.7	-83.5	*Scgb3a1*	-6.7	-8.2	*Retnlg*	-14.0	1.6
*Muc16*	-63.4	-83.4	*Reg3g*	-6.3	-10.5	*Retnla*	-13.7	10.6
*Gp2*	-60.9	-92.3	Xist*	-6.2	-9.8	*Ccl24*	-12.6	3.8
*Krt7*	-60.1	-51.7	*Clec4e*	-5.4	-1.7	*Mmp12*	-10.1	-1.2
*Atp1b1*	-58.6	-56.7	*Slc4a1*	-5.3	-4.6	*AA467197*	-7.6	1.0
*Igf2*	-57.4	-78.0	*Rsad2*	-5.1	-3.5	*AB124611*	-6.3	-2.9
H19*	-57.3	-137.5	*Mmp14*	-4.8	-1.8	*Ldhb*	-6.0	-5.1
*Msln*	-54.2	-7.1	*BC100530*	-4.7	-3.1	*Tarm1*	-5.9	-1.5
*Clic3*	-53.7	-5.9	*Alas2*	-4.1	-3.1	*Itgam*	-5.8	-2.4
*AU021092*	-52.9	-5.7	*Gm5416*	-4.0	-36.8	*Ebi3*	-5.8	-4.6
*BC048546*	-52.9	-5.7	*Gypa*	-4.0	-3.2	*Alox15*	-5.6	3.5

Top 15 developmentally up- and down-regulated genes from purified macrophages of WT mice between three separate age intervals. The fold-changes for these top 15 genes are shown for both WT and *Scnn1b*-Tg mice.

**Table 7:** **Developmentally regulated genes in purified macrophages from** ***Scnn1b-*** **Tg mice**

Gene Name	Fold change: PND 3 vs 0		Gene Name	Fold change: PND 10 vs 3		Gene Name	Fold change: PND 42 vs 10	
	***Scnn1b*** -Tg	WT		***Scnn1b-*** Tg	WT		***Scnn1b*** -Tg	WT
**UP-REGULATED**								
*Irg1*	118.2	8.5	*Ear11*	10.5	39.5	*Rbp4*	69.2	-1.8
*Saa3*	55.5	5.5	*Fabp1*	6.7	2.4	*Spag11b*	21.0	23.8
*Clec4e*	52.9	8.0	*Ccl17*	5.0	9.4	*Fbp1*	15.8	-19.1
*Gpr84*	40.7	3.5	*Ccl24*	4.6	12.2	*Ear11*	14.0	-44.3
*Inhba*	39.3	1.8	*Coro6*	4.3	1.8	*Awat1*	13.4	-3.4
*Gm5416*	32.3	-1.6	*Ch25h*	3.9	3.3	*Bex1*	12.8	-1.5
*Il1f9*	32.2	3.6	*Sorbs3*	3.8	2.4	*Retnla*	10.6	-13.7
*Cxcl3*	26.2	1.9	*Fbp1*	3.8	18.0	*Sox7*	10.0	-1.1
*Slc11a1*	23.2	2.2	*Sftpc*	3.2	1.0	*Lhx2*	9.5	-1.2
*Pla2g7*	23.1	4.3	*Ear5*	3.2	2.4	*Scgb1a1*	8.5	3.2
*Ccrl2*	21.5	4.6	*Map1b*	3.1	-1.1	*Slc1a2*	7.8	1.0
*Mcoln3*	18.6	9.4	*Ffar4*	3.0	1.8	*Arnt2*	7.2	1.0
*Aoah*	18.3	3.9	*Pdk4*	2.9	1.6	*Cpne5*	7.0	18.7
*Slc7a11*	17.6	5.3	*Htr2c*	2.9	2.2	*Scd1*	6.9	1.6
*Cxcl1*	17.0	1.1	*Mamdc2*	2.9	1.4	*Ear5*	6.8	-1.0
**DOWN-REGULATED**								
*Agr2*	-171.4	-223.2	*Stfa3*	-60.6	-2.8	*BC100530*	-9.0	-3.9
*Chad*	-163.4	-105.2	*Saa3*	-51.9	-8.6	*Mmp14*	-7.9	-1.5
H19*	-137.5	-57.3	*Lcn2*	-48.9	-3.8	*Nt5e*	-7.9	-1.3
*Tcf21*	-129.7	-36.3	*Gm5416*	-36.8	-4.0	*Hp*	-7.6	-1.3
*Fmo2*	-119.6	-36.6	*BC100530*	-27.9	-4.7	*Stfa2l1*	-6.5	-2.9
*Muc5b*	-113.5	-83.9	*Stfa2*	-24.4	-2.6	*Irg1*	-6.2	1.0
*AU021092*	-92.4	-52.9	*Chi3l1*	-20.0	-1.7	*Spink2*	-5.9	-5.0
*Gp2*	-92.3	-60.9	*Stfa2l1*	-19.0	-2.8	*Clec4e*	-5.8	2.0
Meg3*	-84.1	-65.4	*S100a9*	-18.9	-1.8	*Cxcl2*	-5.8	-1.9
*Lypd2*	-83.5	-64.7	*Olfm4*	-15.5	-1.5	*Apoc2*	-5.3	-2.9
*Muc16*	-83.4	-63.4	*Asprv1*	-13.8	-1.8	*Apoe*	-5.2	-3.8
*Igf2*	-78.0	-57.4	*Prok2*	-12.3	-1.1	*Ldhb*	-5.1	-6.0
*Acta1*	-76.7	-2.0	*Thbs1*	-12.2	-1.8	*Hilpda*	-5.0	-1.2
*Fhl1*	-72.2	-21.6	*Il1r2*	-12.0	-1.4	*Lpcat2*	-4.9	-1.6
*BC048546*	-71.2	-52.9	*Ifitm1*	-11.2	-1.7	*Sftpc*	-4.8	-1.6

Top 15 developmentally up- and down-regulated genes from purified macrophages of *Scnn1b-*Tg mice between three separate age intervals. The fold-changes for these top 15 genes are shown for both *Scnn1b*-Tg and WT mice.

**Table 8:** **Developmentally regulated Gene Ontology groups in purified macrophages from WT and** ***Scnn1b-*** **Tg** ***mice***

PND 3 vs PND 0		PND 10 vs PND 3		PND 42 vs PND 10	
WT	***Scnn1b*** -Tg	WT	***Scnn1b*** -Tg	WT	***Scnn1b*** -Tg
**UP-REGULATED**					
***GO:0007059 Chromosome segregation***	GO:0019882 Antigen processing and presentation	NONE*	NONE*	GO:0007157 Heterophilic cell-cell adhesion	GO:0032944 Regulation of mononuclear cell proliferation
GO:0000070 Mitotic sister chromatid segregation	***GO:0007059 Chromosome segregation***			NONE*	GO:0070663 Regulation of leukocyte proliferation
GO:0000819 Sister chromatid segregation	GO:0048002 Antigen processing and presentation of peptide antigen				GO:0050670 Regulation of lymphocyte proliferation
GO:0006310 DNA recombination	GO:0071346 Cellular response to interferon-gamma				GO:0045058 T cell selection
GO:0006302 Double-strand break repair	GO:0032615 Interleukin-12 production				GO:0050870 Positive regulation of T cell activation
GO:0007067 Mitosis	GO:0032655 Regulation of interleukin-12 production				GO:0042129 Regulation of T cell proliferation
GO:0006281 DNA repair	GO:0045087 Innate immune response				GO:0006720 Isoprenoid metabolic process
GO:0048285 Organelle fission	GO:0002474 Antigen processing and presentation of peptide antigen via MHC class I				GO:0051251 Positive regulation of lymphocyte activation
GO:0000280 Nuclear division	GO:0032606 Type I interferon production				GO:0048002 Antigen processing and presentation of peptide antigen
GO:0000725 Recombinational repair	GO:0032479 Regulation of type I interferon production				GO:0070661 Leukocyte proliferation
**DOWN-REGULATED**					
***GO:0060541 Respiratory system development***	***GO:0060485 Mesenchyme development***	GO:0035456 Response to interferon-beta	GO:0030595 Leukocyte chemotaxis	GO:0007059 Chromosome segregation	***GO:0007067 Mitosis***
***GO:0030324 Lung development***	***GO:0030324 Lung development***	GO:0035458 Cellular response to interferon-beta	GO:0050900 Leukocyte migration	***GO:0007067 Mitosis***	***GO:0000280 Nuclear division***
***GO:0030323 Respiratory tube development***	***GO0030323 Respiratory tube development***	***GO:0045087 Innate immune response***	***GO:0045087 Innate immune response***	***GO:0000280 Nuclear division***	***GO:0007059 Chromosome segregation***
***GO:0060485 Mesenchyme development***	***GO:0060541 Respiratory system development***	GO:0045851 pH reduction	GO:0006954 Inflammatory response	***GO:0048285 Organelle fission***	***GO:0048285 Organelle fission***
GO:0002009 Morphogenesis of an epithelium	GO:0001657 Ureteric bud development	GO:0051607 Defense response to virus	GO:0006955 Immune response	***GO:0000070 Mitotic sister chromatid segregation***	GO:0000819 Sister chromatid segregation
***GO:0048762 Mesenchymal cell differentiation***	GO:0055123 Digestive system development	GO:0051453 Regulation of intracellular pH	GO:0060326 Cell chemotaxis	***GO:0000819 Sister chromatid segregation***	GO:0033700 Phospholipid efflux
GO:0048729 Tissue morphogenesis	***GO:0048762 Mesenchymal cell differentiation***	GO:0051452 Intracellular pH reduction	GO:0048520 Positive regulation of behavior	GO:0000278 Mitotic cell cycle	***GO:0000070 Mitotic sister chromatid segregation***
GO:0003007 Heart morphogenesis	***GO:0048562 Embryonic organ morphogenesis***	GO:0048525 Negative regulation of viral process	GO:0032103 Positive regulation of response to external stimulus	GO:0000725 Recombinational repair	***GO:0007052 Mitotic spindle organization***
***GO:0048562 Embryonic organ morphogenesis***	GO:0048565 Digestive tract development	GO:0030641 Regulation of cellular pH	GO:0002690 Positive regulation of leukocyte chemotaxis	***GO:0007052 Mitotic spindle organization***	GO:0070301 Cellular response to hydrogen peroxide
GO:0010632 Regulation of epithelial cell migration	GO:2000736 Regulation of stem cell differentiation	GO:0002224 Toll-like receptor signaling pathway	GO:0002687 Positive regulation of leukocyte migration	GO:0000724 Double-strand break repair via homologous recombination	GO:0090068 Positive regulation of cell cycle process

Top ten developmentally up- and down-regulated Gene Ontology groups for purified macrophages in WT and *Scnn1b-*Tg mice between the specified developmental intervals. Gene Ontology groups in common between the WT and *Scnn1b*-Tg line are highlighted by bolded and italicized text. Groups are only listed if FDR <0.1.

*NONE indicates that no groups met the significance threshold (FDR<0.1).

GSEA pathways analysis was more informative than gene-level data to establish the overall trends (Table 8 Additional file 1 Figure S4). In the PND 0–3 interval, WT macrophages exhibited up-regulation of GO pathways involved in cell growth and differentiation, and, interestingly, up-regulation of pathways in WT mice at the later two intervals was minimal. Down-regulated signals during the PND 0–3 interval suggest that these early post-natal macrophages have the ability to respond to signals that are directing normal lung development and differentiation. We cannot rule out the possibility that the macrophage preparations contain a small percentage of epithelial cells, which would confound interpretation. However, epithelial cell contamination was not obvious histologically (data not shown). The strength of down-regulated signals in macrophages for GO lung and respiratory development pathways (Table 8) during this interval suggest that lung epithelium and macrophages use similar signals to coordinate differentiation between the two tissues. Down-regulation of the cell proliferation pathways occurring in the WT and *Scnn1b*-Tg mice at the PND 10–42 period are consistent with the establishment of long-lived, slow proliferating pulmonary macrophage populations during steady state [39, 40]. Significant down-regulation of GO innate immune response pathways in both lines during the PND 3–10 interval points toward the steady-state, mature pulmonary macrophage as relatively quiescent and tolerant toward low danger stimuli. Finally, unlike WT macrophages, *Scnn1b*-Tg macrophages exhibited up-regulation of pathways involved in innate immune responses as expected from their morphologically activated state, with the GSEA signatures being especially robust between the PND 10 and PND 42 (Table 8). Further exploration by directly comparing gene expression between WT and *Scnn1b*-Tg macrophages provided additional insights.

As expected from the PCA, DEGs were identified between WT and *Scnn1b*-Tg macrophages at each time point evaluated, with 432, 394, 166, and 437 DEGs at PND 0, 3, 10, and 42, respectively, using the established significance threshold (FC ≥ 2; FDR ≤ 0.05) (Figure 3c; Additional file 2: Results file S1). As expected from the previous discussion, the top-signaling genes vary across time and represent a variety of biological processes (Table 9). The most significant GO pathways up-regulated by disease at PND 0 are related to muscle cell gene expression, which is difficult to reconcile with the known literature on pulmonary macrophages. However, by PND 3 and onward, significant up-regulation of a variety of inflammatory signatures was observed in the *Scnn1b*-Tg mice compared to WT mice (Table 10). The down-regulation of GO pathways related to mitosis in *Scnn1b*-Tg mice at the earlier time points (PND 3 and PND 10) indicate that *Scnn1b*-Tg pulmonary macrophages are less prolific than their WT counterparts at these early post-natal ages, a shift that is likely a response to the altered cytokine milieu that develops in the *Scnn1b*-Tg mice as a response to the signals that are arising from the bacterial infections and/or necrotic club cells at these time points.

**Table 9:** **Differentially regulated genes between purified macrophages from** ***Scnn1b-*** **Tg and WT mice**

PND 0			PND 3			PND 10			PND 42		
Gene Name	Fold- Change	FDR p-value	Gene Name	Fold-Change	FDR p-value	Gene Name	Fold- Change	FDR p-value	Gene Name	Fold- Change	FDR p-value
**UP-REGULATED**											
*Acta1*	43.4	*9.12E-16*	*Lcn2*	42.6	*4.11E-05*	*Inhba*	25.8	*1.04E-05*	*Ear11*	137.8	*4.70E-06*
*Tnnc2*	37.9	*4.27E-15*	*Stfa3*	35.2	*1.70E-05*	*Mmp14*	22.3	*1.14E-06*	*Fstl1*	85.4	*6.63E-12*
*Csn1s2a*	32.6	*2.11E-06*	*Gm5416*	32.0	*4.46E-05*	*Irg1*	17.1	*9.85E-05*	*Rbp4*	70.5	*3.57E-09*
*Tg*	25.9	*3.53E-03*	*Thbs1*	27.3	*6.67E-06*	*Npy*	15.3	*4.18E-05*	*Fbp1*	67.1	*2.23E-05*
*Csn2*	23.4	*5.58E-05*	*Stfa2*	26.4	*9.55E-05*	*H2-M2*	14.0	*1.58E-09*	*Mfge8*	52.0	*1.38E-11*
*Mylpf*	22.7	*1.19E-07*	*Chi3l1*	20.6	*8.07E-06*	*Slc11a1*	12.8	*5.51E-06*	*Awat1*	51.9	*3.74E-08*
*Scnn1b*	21.5	*2.01E-03*	*Saa3*	20.2	*2.00E-04*	*Clec4e*	12.3	*5.88E-06*	*Arg1*	42.7	*1.17E-05*
*Wap*	19.5	*7.79E-04*	*Inhba*	20.1	*1.74E-05*	*Cxcl2*	11.9	*2.64E-06*	*Inhba*	36.8	*5.26E-07*
*Slc4a1*	17.8	*8.38E-03*	*Cxcl1*	15.8	*2.63E-06*	*Pmp22*	11.0	*1.20E-03*	*Mmp12*	33.9	*7.64E-09*
*Glycam1*	17.7	*3.44E-04*	*Stfa2l1*	15.8	*2.75E-03*	*Ass1*	10.9	*2.25E-05*	*Retnla*	33.8	*3.12E-04*
*Car3*	16.1	*1.90E-03*	*Prok2*	14.3	*4.83E-11*	*Hp*	10.3	*7.72E-06*	*Ppap2b*	27.8	*3.32E-12*
*Rsad2*	16.0	*2.29E-03*	*Plbd1*	14.0	*4.65E-05*	*Mfge8*	10.0	*1.22E-06*	*H2-M2*	25.0	*6.63E-12*
*Csn1s1*	15.9	*1.70E-03*	*Irg1*	13.9	*1.09E-04*	*Cxcl3*	9.7	*2.62E-04*	*Ccl24*	24.2	*1.07E-03*
*Tnnt3*	15.2	*1.58E-08*	Gm10872*	13.9	*7.19E-04*	*Cxcl16*	9.2	*2.64E-06*	*Bex1*	18.9	*2.04E-08*
*Gypa*	14.0	*9.23E-03*	*Cxcl3*	13.6	*2.25E-05*	*Pla2g7*	8.4	*3.15E-04*	*AA467197*	17.4	*2.17E-07*
**DOWN-REGULATED**											
*Gm10473*	-3.8	*5.41E-03*	*Coro6*	-6.1	*4.47E-04*	*Cidec*	-2.7	*1.07E-02*	*Epcam*	-5.2	*4.79E-06*
Gm24049*	-3.6	*2.14E-02*	*Hpgd*	-5.1	*4.44E-04*	*Rab44*	-2.7	*4.02E-04*	*Gal*	-5.1	*6.71E-04*
*6720489N17Rik*	-3.3	*2.23E-02*	*Fabp1*	-5.1	*3.16E-04*	*G0s2*	-2.6	*1.33E-02*	*Tnfsf13b*	-4.8	*6.04E-11*
*Lilra5*	-2.9	*5.01E-04*	*Flt1*	-3.6	*8.97E-06*	*Fam212a*	-2.6	*4.99E-03*	*Dnahc11*	-4.5	*2.76E-08*
*1600002K03Rik*	-2.9	*3.39E-02*	*Kazald1*	-3.4	*2.10E-03*	*Gm5936*	-2.5	*2.25E-02*	*Kazald1*	-4.0	*3.85E-04*
Snora74a*	-2.8	*4.99E-02*	*Gpr34*	-2.9	*6.50E-04*	*Prr5l*	-2.5	*3.11E-02*	*Nt5e*	-4.0	*5.80E-05*
Snora73b*	-2.8	*1.02E-02*	*Slc6a4*	-2.9	*3.94E-04*	*Tmem150b*	-2.4	*9.43E-03*	*Cpne5*	-3.9	*4.10E-04*
*Gdf15*	-2.7	*3.36E-03*	*Gm1966*	-2.9	*4.65E-05*	*Csf3r*	-2.4	*2.37E-02*	*Slc9a2*	-3.8	*1.03E-05*
*Vgf*	-2.7	*3.30E-02*	*Fam212a*	-2.8	*7.44E-04*	*Cd2*	-2.4	*4.82E-03*	*Fam212a*	-3.7	*2.91E-05*
*2010005H15Rik*	-2.7	*3.73E-02*	*Cd2*	-2.8	*3.54E-04*	*Klk8*	-2.4	*1.20E-02*	*Egfem1*	-3.7	*9.82E-05*
*Aqp11*	-2.7	*9.12E-03*	*Klk8*	-2.8	*9.32E-04*	*Kcnh4*	-2.3	*1.19E-02*	*Gpr141*	-3.7	*2.18E-03*
*P2ry13*	-2.6	*7.04E-03*	*Gm11545*	-2.8	*1.37E-02*	*Sec14l2*	-2.2	*7.39E-03*	*Itgad*	-3.5	*1.23E-05*
F630111L10Rik*	-2.6	*1.26E-02*	*Rnase6*	-2.8	*1.17E-02*	*Gm12258*	-2.2	*2.18E-02*	*Iqgap2*	-3.4	*5.72E-06*
*Ch25h*	-2.6	*2.26E-02*	*Trp53i11*	-2.8	*2.74E-02*	*Gsg2*	-2.1	*4.20E-03*	*Pnpla5*	-3.3	*6.36E-06*
*Krt6a*	-2.6	*3.37E-04*	*Cidec*	-2.7	*6.13E-03*	*Arhgef39*	-2.0	*1.36E-02*	*Grap2*	-3.3	*7.52E-04*

Differentially expressed up- and down-regulated (fold-change >2; FDR <0.05) genes between *Scnn1b-*Tg and WT mice from purified macrophages at the four developmental times. Fold-changes are *Scnn1b*-Tg:WT.

**Table 10:** **Differentially regulated Gene Ontology groups from purified macrophages between WT and** ***Scnn1b-*** **Tg mice**

PND 0	PND 3	PND 10	PND 42	PND 42 Germ-free
**UP-REGULATED**				
GO:0006936	GO:0030595	GO:0072593	GO:0009611	GO:0006954
Muscle contraction	Leukocyte chemotaxis	Reactive oxygen species metabolic process	Response to wounding	Inflammatory response
GO:0003012	GO:0032103	GO:0030595	GO:0032103	GO:0009611
Muscle system process	Positive regulation of response to external stimulus	Leukocyte chemotaxis	Positive regulation of response to external stimulus	Response to wounding
GO:0003007	GO:0060326	GO:0060326	GO:0030595	GO:0030595
Heart morphogenesis	Cell chemotaxis	Cell chemotaxis	Leukocyte chemotaxis	Leukocyte chemotaxis
GO:005500	GO:0050900	GO:0060445	GO:0006954	GO:0032103
Striated muscle cell development	Leukocyte migration	Branching involved in salivary gland morphogenesis	Inflammatory response	Positive regulation of response to external stimulus
GO:0060537	GO:0006935	GO:2000379	GO:0071621	GO:0071345
Muscle tissue development	Chemotaxis	Positive regulation of reactive oxygen species metabolic process	Granulocyte chemotaxis	Cellular response to cytokine stimulus
GO:0055001	GO:0071621	GO:0032103	GO:0002687	GO:0034097
Muscle cell development	Granulocyte chemotaxis	Positive regulation of response to external stimulus	Positive regulation of leukocyte migration	Response to cytokine stimulus
GO:0043062	GO:0002687	GO:2000147	GO:0002685	GO:0042330
Extracellular structure organization	Positive regulation of leukocyte migration	Positive regulation of cell motility	Regulation of leukocyte migration	Taxis
GO:0051216	GO:0042330	GO:0050921	GO:0002253	GO:0050714
Cartilage development	Taxis	Positive regulation of chemotaxis	Activation of immune response	Positive regulation of protein secretion
GO:0031032	GO:0050795	GO:2000377	GO:0002757	GO:0006935
Actomyosin structure organization	Regulation of behavior	Regulation of reactive oxygen species metabolic process	Immune response-activating signal transduction	Chemotaxis
GO:0030239	GO:0048520	GO:0002690	GO:0042330	GO:0002685
Myofibril assembly	Positive regulation of behavior	Positive regulation of leukocyte chemotaxis	Taxis	Regulation of leukocyte migration
**DOWN-REGULATED**				
GO:0007186	GO:0006996	GO:0007059	NONE*	NONE*
G-protein coupled receptor signaling pathway	Organelle organization	Chromosome segregation		
GO:0034470	GO:0007059	GO:0000819		
ncRNA processing	Chromosome segregation	Sister chromatid segregation		
GO:0006364	GO:0000819	GO:0000070		
rRNA processing	Sister chromatid segregation	Mitotic sister chromatid segregation		
GO:0016072	GO:0006261	GO:0032465		
rRNA metabolic process	DNA-dependent DNA replication	Regulation of cytokinesis		
GO:0034660	GO:0006302	GO:0006281		
ncRNA metabolic process	Double-strand break repair	DNA repair		
GO:0042254	GO:0000070	GO:0032508		
Ribosome biogenesis	Mitotic sister chromatid segregation	DNA duplex unwinding		
GO:0045076	GO:0007051	GO:0051983		
Regulation of interleukin-2 biosynthetic process	Spindle organization	Regulation of chromosome segregation		
GO:0008033	GO:0051225	GO:0007051		
tRNA processing	Spindle assembly	Spindle organization		
NONE*	GO:0008608	GO:0000280		
	Attachment of spindle microtubules to kinetochore	Nuclear division		
	GO:0000724	GO:0007067		
	Double-strand break repair via homologous recombination	Mitosis		

Top ten differentially up- and down-regulated Gene Ontology groups from purified macrophages between WT and *Scnn1b-*Tg mice at the four developmental stages. Groups are only listed if FDR <0.1.

*NONE indicates that no groups or no additional groups met the significance threshold (FDR <0.1).

#### Custom-annotated pathways allow assessment of disease-specific features

**Figure 4:**
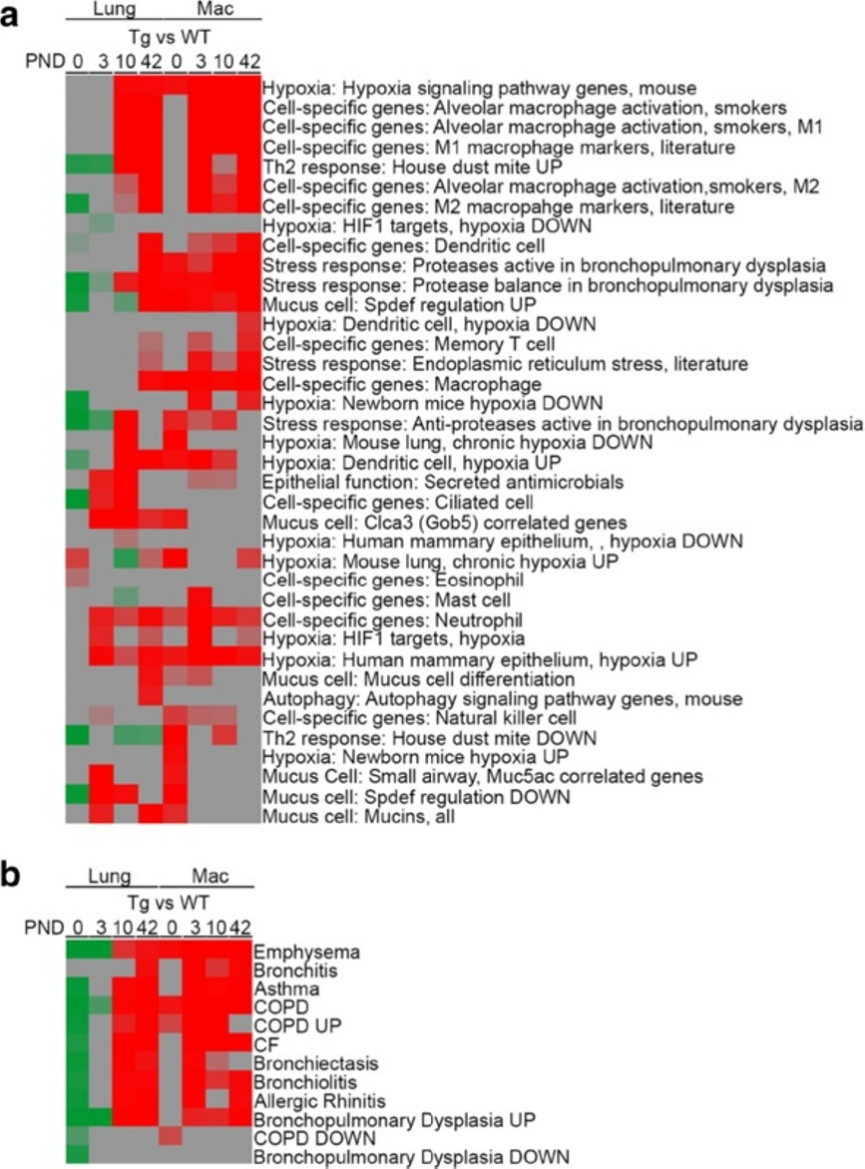
**GSEA using custom gene sets. (a)** Custom gene sets representing putatively relevant processes were used for GSEA (Additional file 4: Results file S2). The FDR values were converted into enrichment scores for clustering by the formula, score = 0.1 – (0.9 *FDR). Red and green indicated up- and down- regulation, respectively. Genes in the custom sets and their sources are described in Additional file 5: Results file S3. **(b)** Heat-map as described in **(a)** for "Respiratory Disease" pathways [41] and COPD-specific pathways [42].

The previously described GSEA analysis, based on publically available GO pathway annotations, provided evidence for activation of relevant biological pathways in both lung and macrophages during the development of disease. However, due to incomplete annotation, GO pathways are not expected to capture all processes specifically associated with lung biology and pulmonary diseases. We hypothesized that additional insights could be provided utilizing custom pathway annotations created to query known, disease-relevant processes. To query our gene expression data for transcriptional events associated with specific pathologic features of the *Scnn1b*-Tg model, customized gene lists were generated that reflected genes hypothesized or known to be involved in the pathogenesis of muco-obstructive lung diseases (Additional file 4: Results file S2 and Additional file 5: Results file S3). Custom pathways were developed either from compilation of literature, e.g., M1 polarized versus M2 polarized pathways, or by selecting genes known to be regulated under specific experimental conditions (e.g.*,* allergen exposure, hypoxia, endoplasmic reticulum stress, autophagy, apoptosis) or cell-specific markers (macrophage activation, ciliated cells, mucous cell, inflammatory cell subsets, secreted antimicrobials) (Figure 4a; Additional file 4: Results file S2). A further group of pathways representing a number of disease-relevant gene expression signatures from human studies as defined by Chowdhary *et. al.*[41] were also queried (Figure 4b; Additional file 4: Results file S2). Literature and database references, as well as details related to the selection of genes in these pathways, are provided (Additional file 5: Results file S3 and Additional file 5: Results file S3 references).

Using these custom annotations, significant GSEA disease-relevant signatures were identified that were tissue/cell specific and time-dependent (Figure 4). In macrophages, substantial evidence for polarization into both M1 and M2 phenotypes was detected. With respect to whole lung, a previously unappreciated up-regulation of ciliated cell- and dendritic cell-specific genes in *Scnn1b*-Tg was identified at PND 3–10 and PND 42, respectively. Mucous cell signatures were consistently found in whole lung at PND 42 that correlated with the expression of epithelial genes previously reported to be induced by SAM-pointed domain–containing Ets-like factor (Spdef), a transcriptional regulator of mucous cell differentiation in mouse and humans [43]. Gene signatures for mucus production were consistent with previous reports [12]. Interestingly, strong up-regulation of Spdef-associated genes was also observed in macrophages at each time point. A mucous cell signature in PND 3 whole lungs, which correlated with up-regulation of genes normally suppressed by Spdef over-expression, was also detected, suggesting a time-dependent activation of alternative pathways. The gene expression pattern of whole lung for secreted antimicrobials correlated with the location and timing of spontaneous infection in *Scnn1b*-Tg mice. Signatures for hypoxia and protease/anti-protease activation were more variable across tissues and time points and more difficult to interpret.

Importantly for the use of this model in the context of human disease, strong up-regulation of human lung disease-specific signatures, including those for chronic obstructive pulmonary disease, were observed in both the lungs (PND 10–42) and macrophages (PND 3–42) (Figure 4b). Specifically, the positive association of DEGs in this study with human DEG disease signatures is seen at the later time points (PND 10 and PND 42). These two time points reflect the establishment and maintenance of the chronic lung disease state in this model. Thus, it is not surprising that they reflect the human tissue better than the earlier time points, since human disease signatures are derived from tissue of patients with established disease, and for the most part, except for BPD, they reflect diseases development that occurs in already mature human lungs.

### Evidence for time-dependent M1 and M2 polarization in *Scnn1b*-Tg macrophages

**Figure 5:**
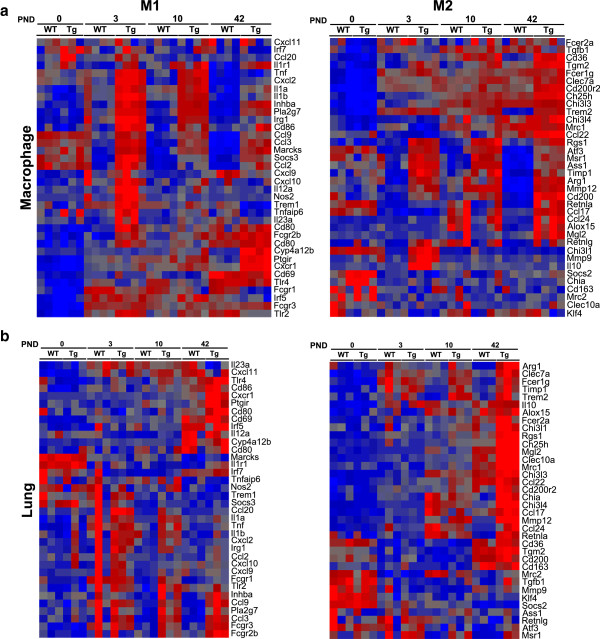
**M1 and M2 DEG signatures in** ***Scnn1b*** **-Tg macrophages and whole lung.** Heat-maps from normalized expression values of M1 and M2 macrophage-activation-related genes (see gene list in Additional file 6: Results file S4 under headings "Macrophage M1 Activation" and "Macrophage M2 Activation") in purified macrophages **(Panel a)** and whole lung **(Panel b)**. Higher and lower expression is represented by red and blue, respectively; with each individual heat-map produced separately (each heat-map has its own unique range of values indicated by dark blue to bright red). The corresponding data can be found in Additional file 6: Results file S4. Each column represents data from one array (n = 3 for lung; n = 4 for other macrophage groups for each genotype), with WT or *Scnn1b*-Tg (Tg) status listed above the columns. Each row represents a single gene.

Because of our interest in the state of pulmonary macrophages in response to disease development, the expression of M1 and M2 markers (Additional file 6: Results file S4) compiled after extensive literature review [44–47] was carefully examined, and the results summarized in heatmaps (Figure 5). The heatmaps highlight the dynamic nature of the macrophage response to airway surface dehydration/defective mucus clearance. Enrichment of both M1 and M2 pathways was evident in PND 3 macrophages, but M1 signatures were particularly robust at PND 3 and M2 signatures particularly robust at PND 42. The M1 signature, while still evident, was clearly different at PND 42 compared to earlier time points, with increased expression of some genes (e.g., *Cxcr1 and Cd69*) and decreased expression of others genes (e.g., *Nos2 and Cxcl2*) compared to PND 3. Similarly, some M2 markers were exclusively high at PND 3 (e.g., *Chi3l1*, *Il10* and *Mmp9),* and others at PND 42 (e.g., *Retnla, Chi3l4, Mrc1*, *Ccl17, Ccl24, Mgl2, Alox15* and *Ccl22*). In addition, some M1 (e.g.*, Cd80, Ccl3 and Socs3*) and M2 (e.g.*, Arg1, Mmp12 and Trem2*) markers were consistently up-regulated during PND 3–42 interval (Figure 5a). The M1 and M2 signatures were also identified globally in whole lung (Figure 5b). The enrichment in M2 markers at PND 42 was confirmed by evaluating protein levels of *Retnla* (Fizz1), *Chi3l3* (YM1), and *Chi3l4* (YM2) in BAL extracts (Additional file 1: Figure S5).

This activation pattern is consistent with the presence of necrotic cells and bacteria in the lungs of the *Scnn1b*-Tg mice at PND 3 and the requirement for macrophages to participate in their clearance via up-regulation of Th1 (M1) responses. Because Th1 responses are known to inhibit Th2 responses [48, 49], including mucous cell metaplastic responses, we hypothesize that the robust Th1 (M1) responses at PND 3 and 10 dampen the Th2-skewed environment normally seen in early post-natal lung development identified in this study and described elsewhere [50]. The shift to M2 polarization after PND 10 reflects the more chronic nature of lung disease, featuring mucus accumulation, but no overt bacterial colonization and normal club cell morphology and function (no necrotic cells). M2 macrophages are known to be critical for defense against atypical fungal and helminth infections [51, 52], and an increase in M2-like alveolar macrophages is characteristic of many inflammatory lung diseases in both humans and mice [20], but the reason for the shift to M2 in our model is not clear. We hypothesize that this (phenomenon) is directly related to the presence of dehydrated mucus with trapped endogenous and exogenous noxious particles and the ability of the macrophages to sense the need to clear this material from the airways. This shift to M2 clearly is expected to have profound consequences, since it is not the normal state in health, and extended long-term activation of M2 macrophages is associated with the establishment of chronic lung disease [53]. Future studies should focus on defining the signals within mucus plugs that drive M2 polarization.

The dynamic nature of both the M1 and M2 signatures, with separate groups of genes activated at different time points, also likely reflects the intrinsic variety of the pulmonary macrophage population [20]. Our sampling technique did not allow a distinction between macrophages residing in localized niches within the lung. Besides the obvious distinction between conducting airway and alveolar spaces, we postulate the existence of several sub-niches where local signals regulate local macrophage phenotypes. These would include areas of localized hypoxia, infections, necrotic/apoptotic cell death, aspiration, and mucus plugging, with the frequency and importance of each of these niches shifting across development [54, 55].

#### Whole lung and macrophages produce independent inflammatory signals

**Figure 6:**
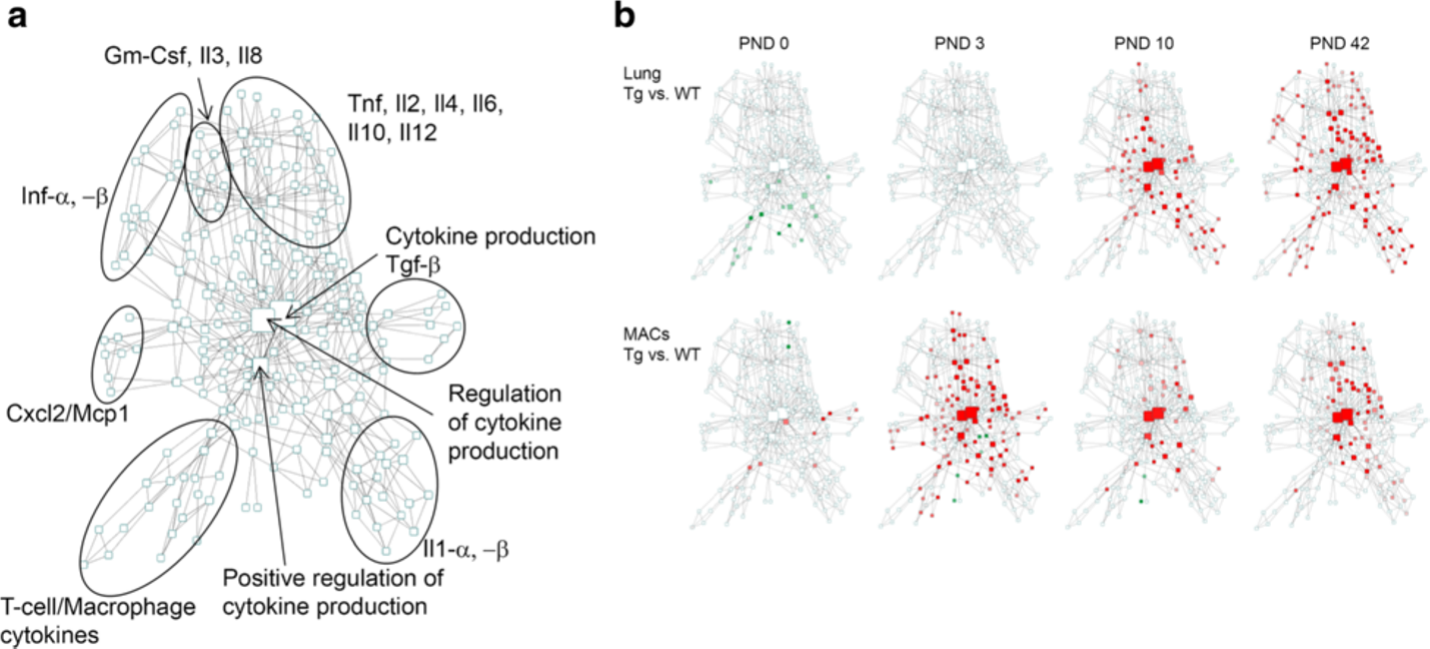
**Dynamics of inflammatory phenotype in the** ***Scnn1b*** **-Tg mouse as represented by cytokine production GO node. (a)** The children nodes of the "Cytokine Production" node of the Gene Ontology biological process were arranged as clusters of connected entities (boxes) according to the hierarchy of GO terms, and visualized in Cytoscape. Each box represents a GO term and associated genes as defined by the GO annotation. Only functional GO terms containing at least 3 annotated genes were included. **(b)** Activation of the "Cytokine Production" GO node in *Scnn1b*-Tg mice in the two tissues studied across time. Red indicates the nodes within this GO cluster that are significantly enriched with annotated genes up-regulated in the *Scnn1b*-Tg mice with the intensity of the color reflecting the significance of the collective shifts of annotated genes to up-regulation (darker = more significant), as reflected by GSEA FDR values. Green represents collective shifts of annotated genes to down-regulation in the *Scnn1b*-Tg mice compared to WT as determined by GSEA.

To further explore the inflammatory signals originating from, or possibly leading to, the M1 and M2 polarization patterns, the "cytokine production" node within Gene Ontology Biological Process was scrutinized (Figure 6a). At PND 0, the only signs of activation of this node were in macrophages (Figure 6b; Additional file 3: Table S3; Additional file 1: Figure S6; Additional file 7: Results file S5). Genes in this GO node were not activated in whole lung until PND 10 with signals increasing further at PND 42 (Figure 6b; Additional file 3: Table S3). While this analysis shows robust activation of the GO node related to *Scnn1b*-Tg expression, careful gene-level evaluation of these signatures revealed that they were primarily derived from either whole lung, e.g.*, Cxcl5, Chia,* and *Ltf*, or macrophages (many), with only a few genes producing signal from both tissues, e.g.*, Ccl3*, *Chi3l1*, and *Tnfrsf*9 (Additional file 3: Table S3). Overall, macrophages exhibited more robust signals than whole lung, with both up-regulated and a small number of down-regulated genes identified. Because we had seen activation of a number of macrophage markers in whole lung (Additional file 3: Table S1), we hypothesized that much of the signal detected from whole lung originated from the activated macrophage population. However, it is clear from our data (Additional file 3: Table S3) that for most DEGs, the epithelial/parenchymal compartment and the macrophages uniquely contribute to the inflammatory signaling in response to airway mucus obstruction. Within each tissue, as for the M1 and M2 genes (Figure 5), DEGs tended to differ across time, e.g.*,* genes robustly signaling in macrophages at PND 0, which were enriched for KEGG "pancreatic cancer" pathways containing *Tgfb2* (not shown), were substantially different than those signaling at PND 3 (enriched for KEGG pathways containing *Il6* and *Tnf* such as those involved in NOD-like and Toll-like receptor signaling), and those signaling at PND 42 (enriched for KEGG pathway "chemokine signaling" containing *Ccr7, Ccr5*, and *Arrb1*). While a detailed analysis of all of the potential mechanistic insights provided by this report is beyond the scope of this publication, this study provides a framework for future efforts directed at individual signaling molecules and further highlights the usefulness of this model for a variety of pulmonary disease research questions.

#### *Scnn1b*-Tg macrophage gene signatures were comparable but more robust in GF vs. SPF environment

**Figure 7:**
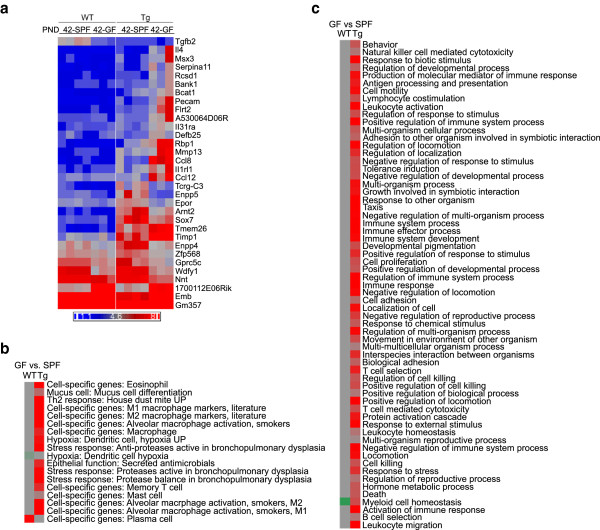
**Gene expression patterns in germ-free (GF) macrophages compared to specific-pathogen free (SPF) environments. (a)** Unsupervised hierarchical clustering of the small set of DEGs that are either differentially expressed between SPF and GF WT macrophages (shown on left) or between SPF and GF *Scnn1b*-Tg macrophages (shown on right) purified from PND 42. Gene symbols are shown to the right. Each column represents expression from a single microarray (n = 3 for GF; n = 4 for SPF). Filtering for DEGs and colors are as described as in Figure 2. GSEA analyses for custom gene-set **(b)** and multiple immune system pathway **(c)** and Gene Ontology annotations comparing SPF versus GF WT macrophages (left column) or SPF versus GF *Scnn1b*-Tg macrophages (right column). Color coding and significance parameters were as in Figure 4. The corresponding data can be found in Additional file 8: Results file S6.

GF macrophages from either WT or *Scnn1b*-Tg mice were very similar compared to their SPF counterparts (Figure 3c), and only 32 genes were differentially expressed across the four groups at the selected significance threshold (Figure 7a). This result is significant because it demonstrates that macrophage activation, at least in the chronic disease state present at PND 42, is not dependent upon the presence of microbes or microbe-derived products, consistent with our previous discussion. By extension, macrophage activation at this stage must be in response to the consequence of epithelial transgene overexpression, namely, airway surface dehydration and the resultant mucus stasis. The gene expression findings are consistent with previous histological analysis in the GF *Scnn1b*-Tg colony [13] and reinforce our hypothesis that, if airway surfaces are dehydrated, static mucus itself and/or abiotic materials concentrated in dehydrated mucus, trigger and maintain lung inflammation and airway remodeling.

While the differential gene expression in the GF macrophages was qualitatively similar between the GF and SPF *Scnn1b*-Tg macrophages (Figure 3; Additional file 1: Figure S7a), the quantitative fold-change was higher in GF compared to SPF *Scnn1b*-Tg mice, and GSEA analysis showed a more robust up-regulation of custom gene set (Figure 7b; Additional file 1: Figure S7a) and multiple immune system (Figure 7c) pathways in macrophages from GF compared to SPF *Scnn1b*-Tg mice (Additional file 8: Results file S6). We speculate that the absence of environmental bacteria and their products altered the homeostatic mechanisms that normally prevent excessive activation of resident macrophages, predisposing GF mice to exaggerated responses to external challenges, including airway surface dehydration (Additional file 1: Figure S7b). These findings are congruent with excessive activation as a key feature of gut inflammatory responses in mice raised in germ-free conditions and they highlight the role of endogenous microflora in establishment of immune homeostasis in the lung as well [56].

## Conclusion

The gene expression analyses reported here provide a global appreciation for the complexity of the development of lung disease in the *Scnn1b*-Tg model. Importantly, the initiating stimulus in the model, i.e., mucus dehydration, is present during early postnatal life, allowing unique investigations into the relationships between a defined disease stimulus (airway surface dehydration) and postnatal lung development. This feature, which leads to emphysema, provides a setting to study the pathogenic crosstalk between mucus obstructed and inflamed airways and alveoli, processes that are likely important in children with BPD, CF, and COPD-like pediatric diseases. The Th1 airway inflammation produced as part of the "insult" transmitted from the airways to the alveolar compartments is a likely culprit in the *Scnn1b*-Tg mice. Our studies also reveal macrophages as early responders to airway mucus stasis and as possible communicators of inflammatory signals from the airways to the alveoli.

In contrast, the gene expression changes identified during the chronic stage of disease are more reflective of typical adult onset muco-obstructive lung diseases. Moving forward, separating the two phase of disease will be necessary to define pathogenic mechanisms. The complexity of gene signatures additionally point toward the necessity of considering cell-specific, and even site-specific, signals in future studies. Further evaluation of cell-specific lineages, including those of airway epithelial cells and macrophages purified from different lung compartments (e.g.*,* airways *vs* alveolar, mucus embedded *vs* free), will be invaluable to generate an integrated picture of the pathways leading to lung disease in this model and likely inform translational therapeutic efforts.

### Availability of supporting data

Data Repository: Please use the following URL in order to access our private GEO database. " http://www.ncbi.nlm.nih.gov/geo/query/acc.cgi?acc=GSE47551".

Additional Supporting Data Files: This file summarizes the information about supplemental files associated with the manuscript titled: "Gene Expression in Whole Lung and Pulmonary Macrophages Reflects the Dynamic Pathology Associated with Airway Surface Dehydration".

## Authors’ information

Co-senior authors: Wanda K O’Neal and Richard C Boucher.

## Electronic supplementary material

Additional file 1: Figure S1: Principal component analysis (PCA). PCA of gene expression from WT and *Scnn1b*-Tg whole lung and purified BAL macrophages. **Figure S2.** Lung Gene Set Enrichment Analysis (GSEA). Heatmap representing top level GO and more stringent GSEA enrichment signals in whole lung between WT and *Scnn1b*-Tg mice at the four times studied. To fit the result into a legible figure, only top level (1 and 2 levels down from the root vocabulary term) GO biological processes and more stringent enrichment score were used as a functional annotation for this figure (Additional file 4: Results file S2). Results were considered significant when FDR < 1%. The FDR values were converted into enrichment scores by the formula: score = 0.01 – (0.9 * FDR). Red and green indicate up- and down- regulation, respectively. **Figure S3.** Macrophage Purification. Representative images showing (a) freshly isolated, unprocessed cells from BALF, (b) purified Ly6G-negative macrophages, and (c) Ly6G positive cells after removal of the Ly6G negative cells shown in (b). **Figure S4.** GSEA from purified macrophages. Heatmap representing top level GO signals in macrophages between WT and *Scnn1b*-Tg mice at the four times studied (Additional file 4: Results file S2). Details of the analysis are provided in legend to Additional file 5: Figure S2. **Figure S5.** Western blot analyses for M2 macrophage activation markers (Chi3l3, Chi3l4 and Retnla) on protein extracts from purified macrophages from PND 42 WT and *Scnn1b*-Tg mice. **Figure S6.** Heat-map of fold changes between *Scnn1b*-Tg and WT mice across tissues of expressed genes (Additional file 7: Results file S5) in the "Cytokine Production" GO node, whose mean log2 intensities are greater than 5 in at least one sample group in whole lung or macrophages. **Figure S7.** M1 and M2 macrophage-activation gene signatures in germ-free (GF) and specific pathogen free (SPF) mice. (PDF 3 MB)

Additional file 2: Results file S1: This excel file contains tables of log2 normalized intensity values for named genes used to generate hierarchical clustering heat-maps in figures indicated by the name of the tables. The original excel file for further analyses will be made available in the supplementary section. To generate the heat-maps, choose one of the 3 gene identifier columns, e.g. "gene symbol" and save the spreadsheet table as tab-delimited text file as input to any software tool designed to clustering microarray data. "Lung_Fig1b_time_cluster" worksheet (for Figure 1b), "Lung_Fig2_BvsWT_cluster" worksheet (for Figure 2), "MAC_Fig3b_time_cluster" worksheet (for Figure 3b), "MAC_Fig3c_BvsWT_cluster" worksheet (for Figure 3c). (XLS 8 MB)

Additional file 3: Table S1: Functional and Gene Ontology Terms associated with the differentially expressed genes between *Scnn1b*-Tg and WT whole lung as outlined in Table 3. Functional categories are based upon author annotations from a literature review. **Table S2.** Percent Cell Counts (Percentage) on Purified Macrophage Fractions. **Table S3.** Top cytokine signaling gene level table corresponding to the "Cytokine Signaling" Gene Ontology pathway results for differential gene expression in whole lung and macrophages comparing *Scnn1b*-Tg mice to WT at the time points indicated. Genes are grouped into categories based upon the characteristics defined in the headings. Genes are only shown if the fold-change was ±2-fold for any condition. Light red indicates up-regulation in *Scnn1b*-Tg compared to WT. Green indicates down-regulation. (PDF 114 KB)

Additional file 4: Results file S2: This excel file contains tables of converted enrichment significance score from GSEA results for clustering analysis. The names of the individual worksheets indicate corresponding figures (Figure 4a and b; Figures S2 and S4) that show the clusters based on the specific data. For each table, GSEA results from relevant individual comparisons were extracted using a specified FDR cutoff, *T* (e.g. 0.1 or 0.01) in at least one of the comparisons. The pooled FDR values (*F*) were then converted to the enrichment score (*S*) by the following formula: *S* = *T* – (0.9 * *F*) contained in these tables for clustering. (XLSX 21 KB)

Additional file 5: Results file S3: This excel file containing custom gene sets using in the GSEA analysis described in the manuscript. The supporting files containing literature reference can be found in Additional file 5: Results file S3 References. The format is in .gmt as documented by GSEA ( http://www.broadinstitute.org/gsea/). To use them in GSEA analysis, export the table from Excel as tab-delimited text file, then rename the text file with .txt extension to .gmt file, and use as GSEA input according to GSEA user guide. (XLSX 144 KB)

Additional file 6: Results file S4: This excel file containing expression values of M1 and M2 custom gene set in macrophages and whole lung that were used to generate Figure 5 and Additional file 1: Figure S7. (XLSX 26 KB)

Additional file 7: Results file S5: This excel file contains data tables for Figure 6. For each comparison between *Scnn1b*-Tg vs WT, GSEA enrichment summary tables (consult http://www.broadinstitute.org/gsea/ documentation for output format) GO biological process terms branching off from "Cytokine production" were copied into each spreadsheet table as indicated in the table name. To regenerate the results, GO terms were displayed as connected networks by Cytoscape ( http://www.cytoscape.org/) and the FDR values were used to convert to enrichment scores described above, from each GSEA summary table saved as tab-delimited text files were used to decorate the GO term nodes as color intensities. The fold change values for "Cytokine production" genes were filtered at minimal of 1.5 fold change between *Scnn1b*-Tg and WT mice for at least 1 time point, then used for clustering in Additional file 1: Figure S6 are in "CytoGene_fold_change_1.5" worksheet. (PDF 781 KB)

Additional file 8: Results file S6: This excel file contains log2 normalized intensity values of genes in macrophages from GF and SPF conditions. This data was used to generate Figure 7. (XLSX 20 KB)
