# Supplementary material for: Gene expression in whole lung and pulmonary macrophages reflects the dynamic pathology associated with airway surface dehydration
Source: BMC Genomics. 2014 Sep 10;15(1):726. doi: 10.1186/1471-2164-15-726 (PMC4247008; doi:10.1186/1471-2164-15-726)
Supplement: Supplementary file 1 — Additional file 1: Figure S1: Principal component analysis (PCA). PCA of gene expression from WT and Scnn1b-Tg whole lung and purified BAL macrophages. Figure S2. Lung Gene Set Enrichment Analysis (GSEA). Heatmap representing top level GO and more stringent GSEA enrichment signals in whole lung between WT and Scnn1b-Tg mice at the four times studied. To fit the result into a legible figure, only top level (1 and 2 levels down from the root vocabulary term) GO biological processes and more stringent enrichment score were used as a functional annotation for this figure (Additional file 4: Results file S2). Results were considered significant when FDR < 1%. The FDR values were converted into enrichment scores by the formula: score = 0.01 – (0.9 * FDR). Red and green indicate up- and down- regulation, respectively. Figure S3. Macrophage Purification. Representative images showing (a) freshly isolated, unprocessed cells from BALF, (b) purified Ly6G-negative macrophages, and (c) Ly6G positive cells after removal of the Ly6G negative cells shown in (b). Figure S4. GSEA from purified macrophages. Heatmap representing top level GO signals in macrophages between WT and Scnn1b-Tg mice at the four times studied (Additional file 4: Results file S2). Details of the analysis are provided in legend to Additional file 5: Figure S2. Figure S5. Western blot analyses for M2 macrophage activation markers (Chi3l3, Chi3l4 and Retnla) on protein extracts from purified macrophages from PND 42 WT and Scnn1b-Tg mice. Figure S6. Heat-map of fold changes between Scnn1b-Tg and WT mice across tissues of expressed genes (Additional file 7: Results file S5) in the "Cytokine Production" GO node, whose mean log2 intensities are greater than 5 in at least one sample group in whole lung or macrophages. Figure S7. M1 and M2 macrophage-activation gene signatures in germ-free (GF) and specific pathogen free (SPF) mice. (PDF 3 MB) [file 12864_2014_6683_MOESM1_ESM.pdf]

Figure S1

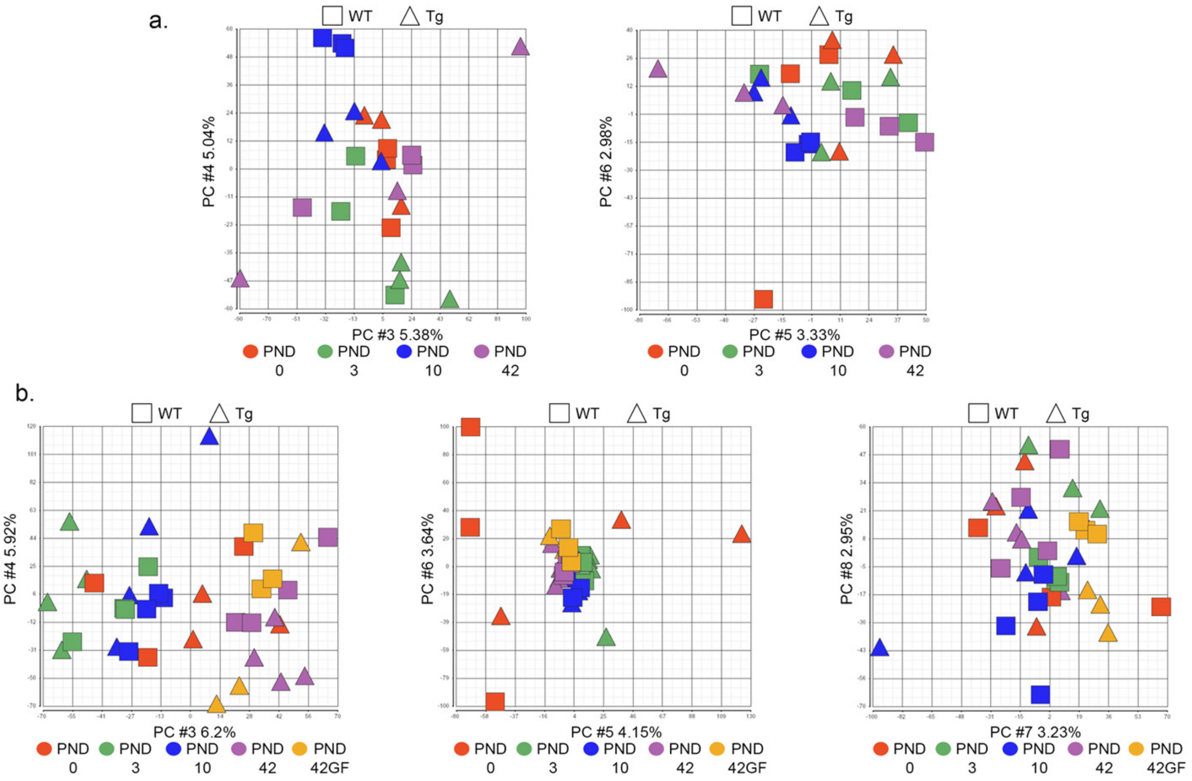

Figure S1. Principal component analysis (PCA). PCA of gene expression from WT and Scnn1b-Tg whole lung and purified BAL macrophages at PND 0, 3, 10, and 42 plotted in two-dimensional space for the selected groups of principal components (PCs; shown on the X and Y axis). (a) PCA of gene expression from WT and Scnn1b-Tg whole lungs using PC #3 and 4 (left) and PC #5 and 6 (right). (b) PCA of gene expression for isolated macrophages from WT and Scnn1b-Tg mice for PCAs as indicated on the axis. Squares = WT; Triangles = Scnn1b-Tg. Age is designated by color: PND0 (red), PND 3 (green), PND 10 (blue), and PND42 (purple). PND 42 macrophages from germ-free (GF) mice are orange. Each symbol represents the results of a single microarray, which for whole lung represents pools of individual mice as described in the methods.

Figure S2

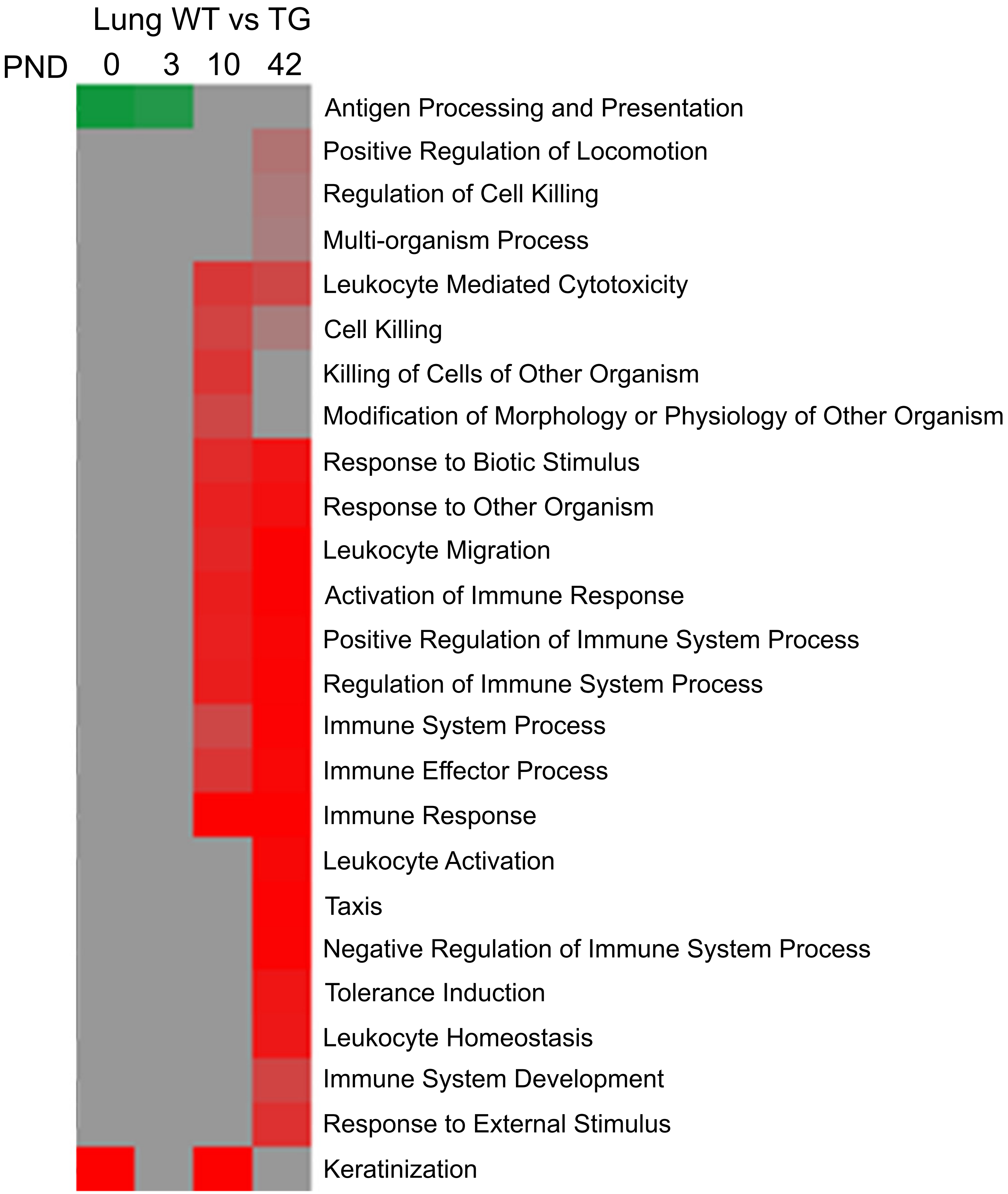
Lung WT vs TG
PND 0 3 10 42
Antigen Processing and Presentation
Positive Regulation of Locomotion
Regulation of Cell Killing
Multi-organism Process
Leukocyte Mediated Cytotoxicity
Cell Killing
Killing of Cells of Other Organism
Modification of Morphology or Physiology of Other Organism
Response to Biotic Stimulus
Response to Other Organism
Leukocyte Migration
Activation of Immune Response
Positive Regulation of Immune System Process
Regulation of Immune System Process
Immune System Process
Immune Effector Process
Immune Response
Leukocyte Activation
Taxis
Negative Regulation of Immune System Process
Tolerance Induction
Leukocyte Homeostasis
Immune System Development
Response to External Stimulus
Keratinization

Figure S3

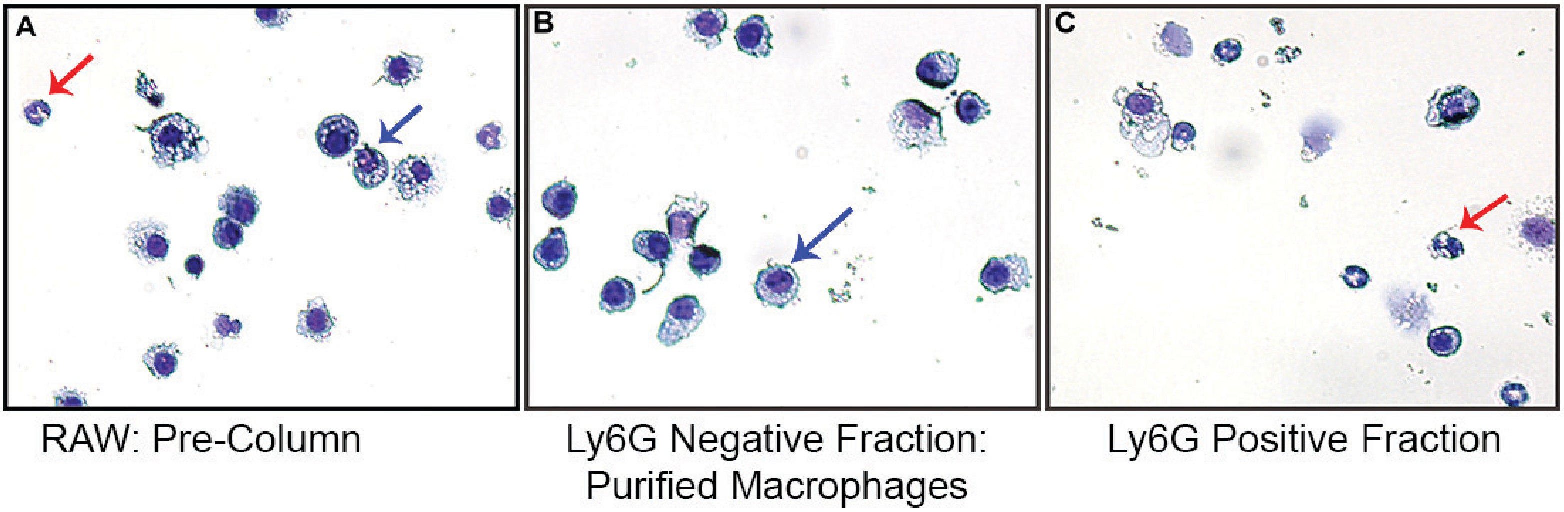

Red and blue arrows indicate representative polymorphonuclear cells [neutrophils; only seen in (a and c)] and macrophages, respectively.

Figure S4

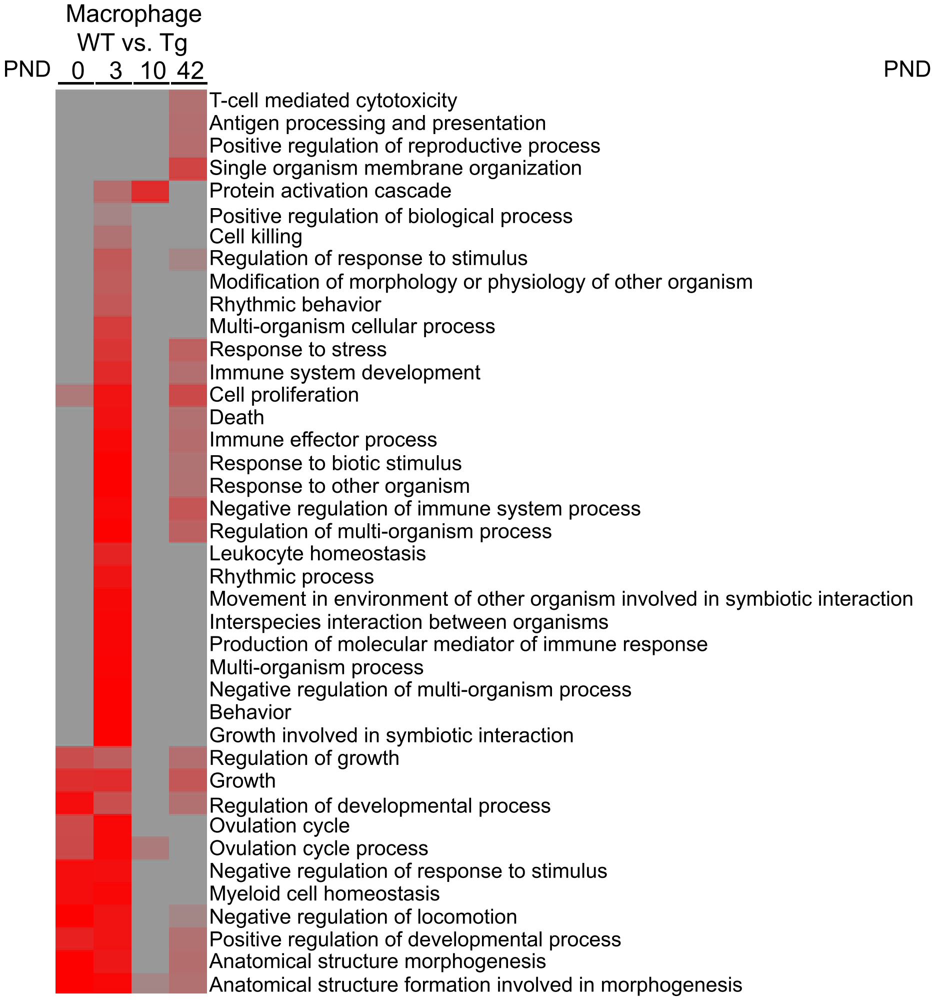

Macrophage
WT vs. Tg
PND
0
3
10
42
T-cell mediated cytotoxicity
Antigen processing and presentation
Positive regulation of reproductive process
Single organism membrane organization
Protein activation cascade
Positive regulation of biological process
Cell killing
Regulation of response to stimulus
Modification of morphology or physiology of other organism
Rhythmic behavior
Multi-organism cellular process
Response to stress
Immune system development
Cell proliferation
Death
Immune effector process
Response to biotic stimulus
Response to other organism
Negative regulation of immune system process
Regulation of multi-organism process
Leukocyte homeostasis
Rhythmic process
Movement in environment of other organism involved in symbiotic interaction
Interspecies interaction between organisms
Production of molecular mediator of immune response
Multi-organism process
Negative regulation of multi-organism process
Behavior
Growth involved in symbiotic interaction
Regulation of growth
Growth
Regulation of developmental process
Ovulation cycle
Ovulation cycle process
Negative regulation of response to stimulus
Myeloid cell homeostasis
Negative regulation of locomotion
Positive regulation of developmental process
Anatomical structure morphogenesis
Anatomical structure formation involved in morphogenesis

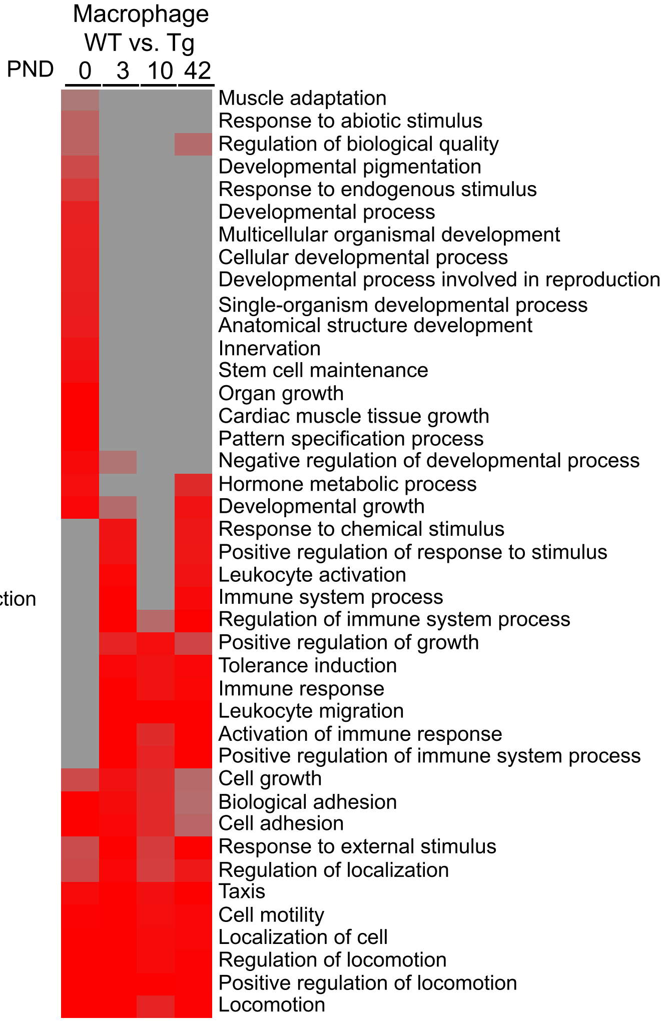

Macrophage
WT vs. Tg
PND
0
3
10
42
Muscle adaptation
Response to abiotic stimulus
Regulation of biological quality
Developmental pigmentation
Response to endogenous stimulus
Developmental process
Multicellular organismal development
Cellular developmental process
Developmental process involved in reproduction
Single-organism developmental process
Anatomical structure development
Innervation
Stem cell maintenance
Organ growth
Cardiac muscle tissue growth
Pattern specification process
Negative regulation of developmental process
Hormone metabolic process
Developmental growth
Response to chemical stimulus
Positive regulation of response to stimulus
Leukocyte activation
Immune system process
Regulation of immune system process
Positive regulation of growth
Tolerance induction
Immune response
Leukocyte migration
Activation of immune response
Positive regulation of immune system process
Cell growth
Biological adhesion
Cell adhesion
Response to external stimulus
Regulation of localization
Taxis
Cell motility
Localization of cell
Regulation of locomotion
Positive regulation of locomotion
Locomotion

Figure S5

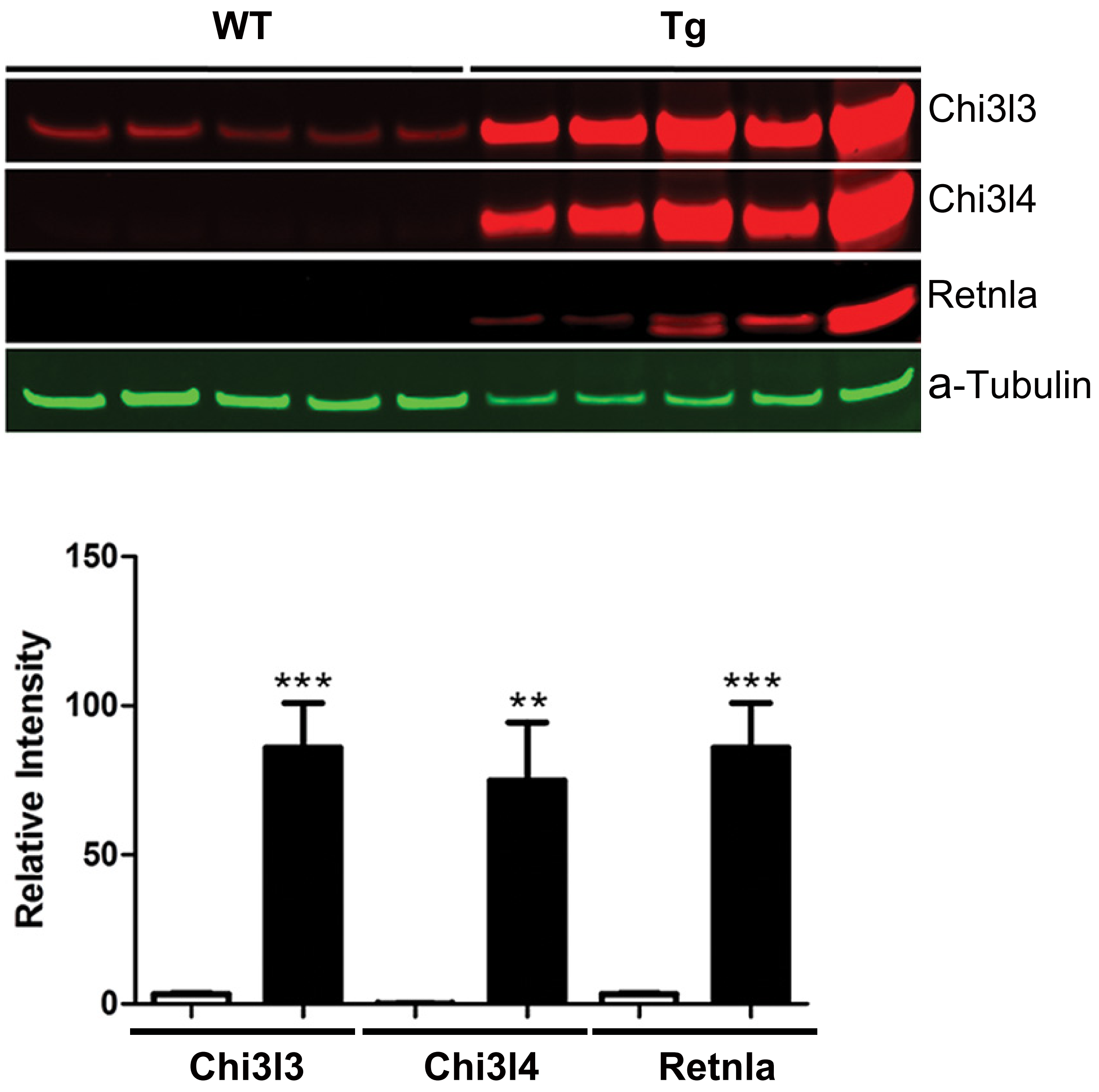

The graph shows the densitometric quantification of the western data. ** = p < 0.01; *** = p < 0.001.

Figure S6

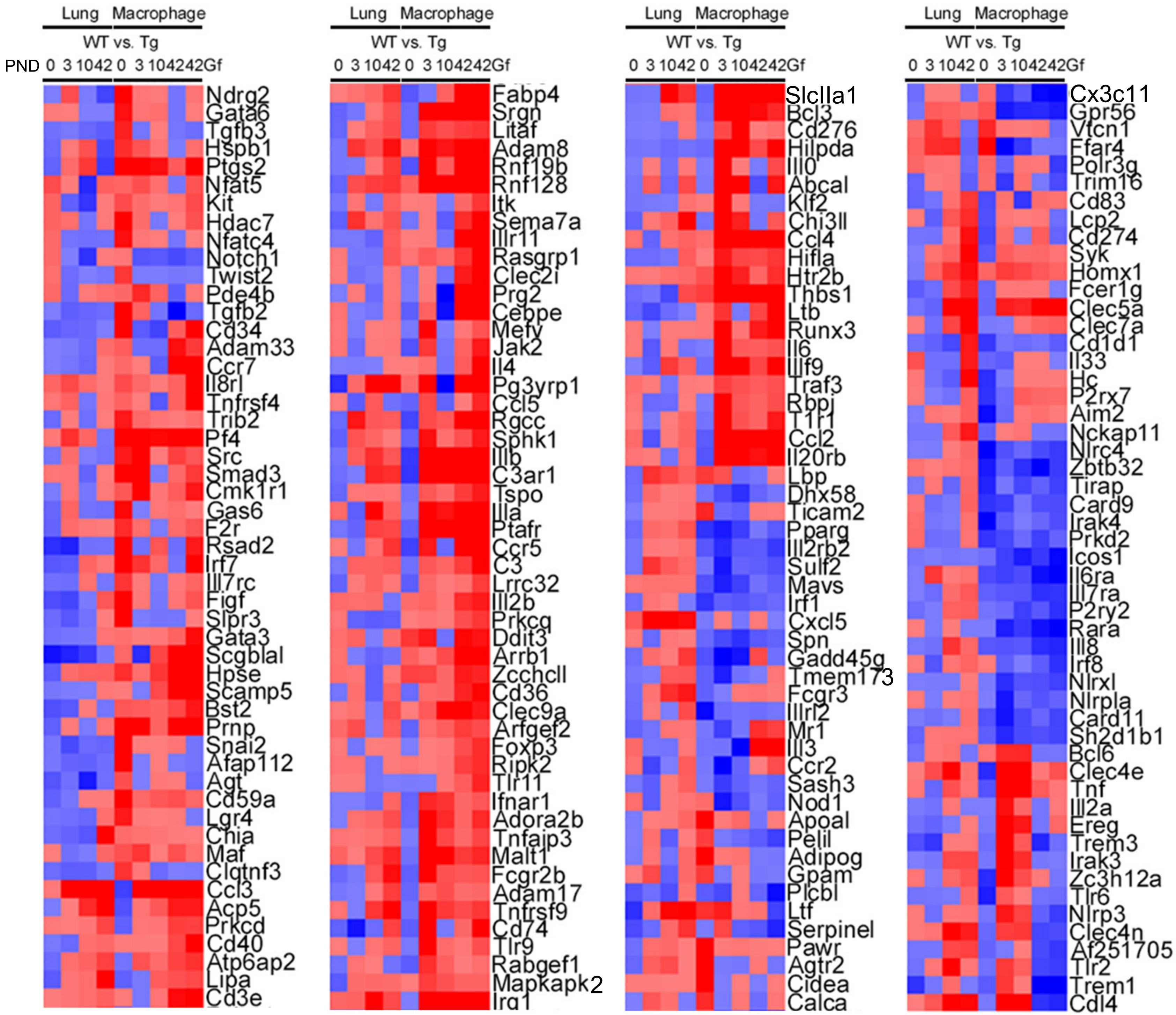
Lung Macrophage
WT vs. Tg
PND 0 3 10 42 0 3 10 42 42Gf
Ndrg2
Gata6
Tgfb3
Hspb1
Ptgs2
Nfat5
Kit
Hdac7
Nfatc4
Notch1
Twist2
Pde4b
Tgfb2
Cd34
Adam33
Ccr7
Il8rl
Tnfrsf4
Trib2
Pf4
Src
Smad3
Cmk1r1
Gas6
F2r
Rsad2
Irf7
Il7rc
Figf
Slpr3
Gata3
Scgblal
Hpse
Scamp5
Bst2
Prnp
Snai2
Afap112
Agt
Cd59a
Lgr4
Chia
Maf
Clqtnf3
Ccl3
Acp5
Prkcd
Cd40
Atp6ap2
Lipa
Cd3e
Lung Macrophage
WT vs. Tg
0 3 10 42 0 3 10 42 42Gf
Fabp4
Srgn
Litaf
Adam8
Rnf19b
Rnf128
Itk
Sema7a
Illr11
Rasgrp1
Clec2i
Prg2
Cebpe
Mefv
Jak2
Il4
Pg3yrp1
Ccl5
Rgcc
Sphk1
Illb
C3ar1
Tspo
Illa
Ptafr
Ccr5
C3
Lrrc32
Ill2b
Prkcq
Ddit3
Arrb1
Zcchcll
Cd36
Clec9a
Arfgef2
Foxp3
Ripk2
Tlr11
Ifnar1
Adora2b
Tnfaip3
Malt1
Fcgr2b
Adam17
Tnfrsf9
Cd74
Tlr9
Rabgef1
Mapkapk2
Irg1
Lung Macrophage
WT vs. Tg
0 3 10 42 0 3 10 42 42Gf
SlcIIa1
Bcl3
Cd276
Hilpda
Ill0
Abcal
Klf2
Chi3ll
Ccl4
Hifla
Htr2b
Thbs1
Ltb
Runx3
Il6
Illf9
Traf3
Rbpj
T1r1
Ccl2
Il20rb
Lbp
Dhx58
Ticam2
Pparg
Ill2rb2
Sulf2
Mavs
Irf1
Cxcl5
Spn
Gadd45g
Tmem173
Fcgr3
Illrl2
Mr1
Ill3
Ccr2
Sash3
Nod1
Apoal
Pelil
Adipog
Gpam
Plcbl
Ltf
Serpinel
Pawr
Agtr2
Cidea
Calca
Lung Macrophage
WT vs. Tg
0 3 10 42 0 3 10 42 42Gf
Cx3c11
Gpr56
Vtcn1
Ffar4
Polr3g
Trim16
Cd83
Lcp2
Cd274
Syk
Homx1
Fcer1g
Clec5a
Clec7a
Cd1d1
Il33
Hc
P2rx7
Aim2
Nckap11
Nlrc4
Zbtb32
Tirap
Card9
Irak4
Prkd2
Icos1
Il6ra
Ill7ra
P2ry2
Rara
Ill8
Irf8
Nlrxl
Nlrpla
Card11
Sh2d1b1
Bcl6
Clec4e
Tnf
Ill2a
Ereg
Trem3
Irak3
Zc3h12a
Tlr6
Nlrp3
Clec4n
Af251705
Tlr2
Trem1
Cdl4

Figure S7

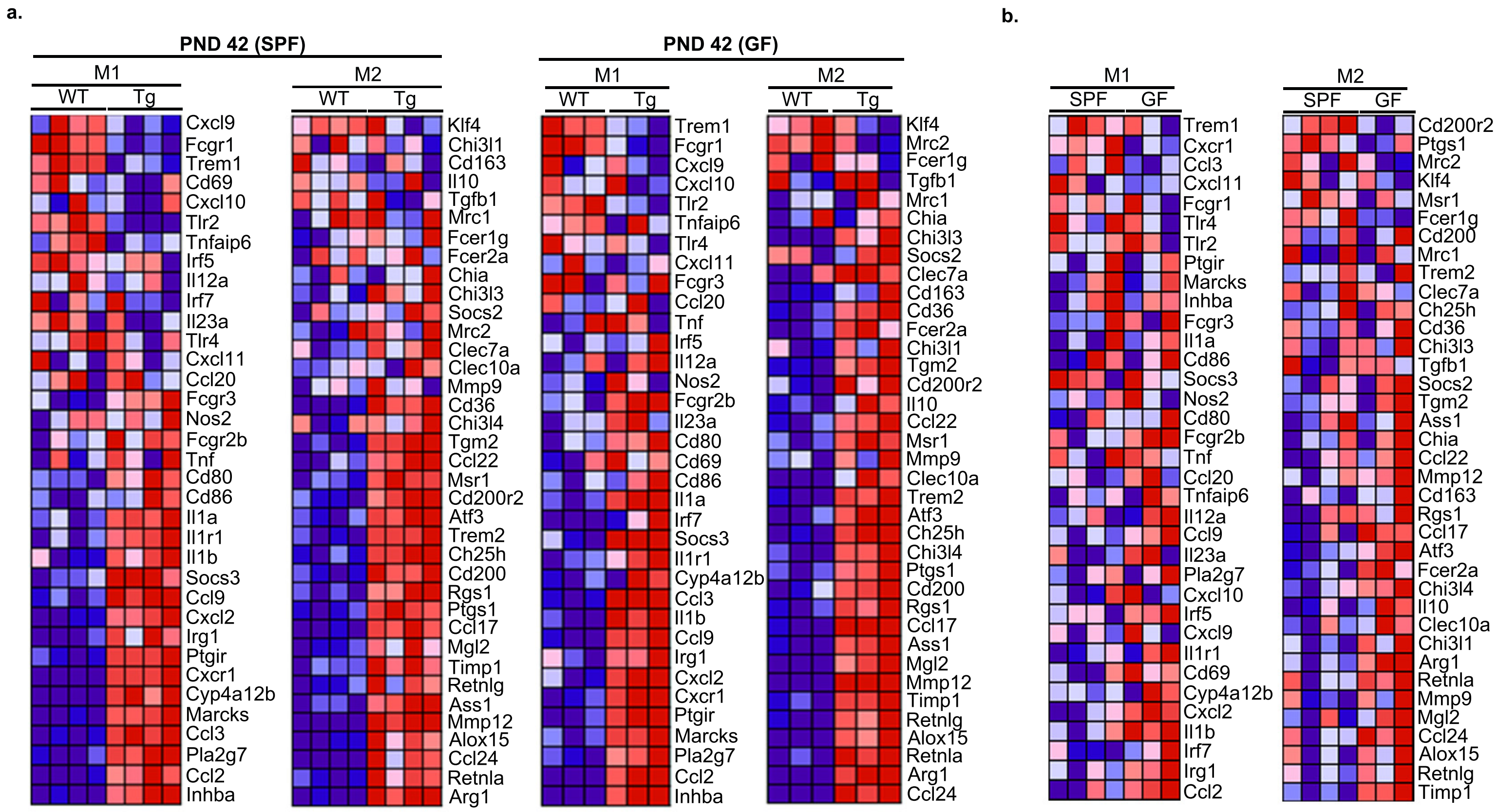

Heat-maps from normalized expression values of M1 and M2 macrophage-activation-related genes (see gene list in Additional file 6: Results file S3 under headings “Macrophage M1 Activation” and “Macrophage M2 Activation”). Higher and lower expression is represented by red and blue, respectively; with each individual heat-map produced separately (each heat-map has its own unique range of values indicated by dark blue to bright red). The corresponding data can be found in Additional file 7: Results file S4. Each column represents data from one array (n = 3 for GF macrophages and lung; n = 4 for SPF macrophage groups for each genotype), with WT or Scnn1b-Tg (Tg) status listed above the columns. Each row represents a single gene.
