# Supplementary material for: Gene expression in whole lung and pulmonary macrophages reflects the dynamic pathology associated with airway surface dehydration
Source: BMC Genomics. 2014 Sep 10;15(1):726. doi: 10.1186/1471-2164-15-726 (PMC4247008; doi:10.1186/1471-2164-15-726)
Supplement: Supplementary file 3 — Additional file 3: Table S1: Functional and Gene Ontology Terms associated with the differentially expressed genes between Scnn1b-Tg and WT whole lung as outlined in Table 3. Functional categories are based upon author annotations from a literature review. Table S2. Percent Cell Counts (Percentage) on Purified Macrophage Fractions. Table S3. Top cytokine signaling gene level table corresponding to the "Cytokine Signaling" Gene Ontology pathway results for differential gene expression in whole lung and macrophages comparing Scnn1b-Tg mice to WT at the time points indicated. Genes are grouped into categories based upon the characteristics defined in the headings. Genes are only shown if the fold-change was ±2-fold for any condition. Light red indicates up-regulation in Scnn1b-Tg compared to WT. Green indicates down-regulation. (PDF 114 KB) [file 12864_2014_6683_MOESM3_ESM.pdf]

**Table 1. Functional and Gene Ontology Terms associated with the differentially expressed genes between *Scnn1b*-Tg and WT whole lung as outlined in main text Table 4. Functional categories are based upon author annotations from a literature review.**						
**Gene name**	**Functional categories**[1]		**Gene ID**	***GO Terms***	**Brief description**	***References***
9930013 L23Rik	6		80982		Protein: KIAA1199; deafness gene; hyaluronan binding protein	*(1)*
Muc5ac	1	2	17833		Secreted airway mucin known to be up-regulated in *Scnn1b*-Tg mice.	*(2)*
Gm22843	6		-		Potassium channel homologue	*None*
Phxr4	6		18689		No known function. Originally cloned from mouse spleen.	*(3)*
Awat1	5	6	245533	*GO:0006629|lipid metabolic process*	Esterifies long chain (wax) alcohols to produce wax esters.	*(4)*
Tm4sf19	6		277203		Involved in endocrine pancreatic differentiation.	*(5)*
Ear11	3	4	93726	*GO:0006935|chemotaxis*	Granule-derived secretory protein; alarmin that activates TLR2; alerts adaptive immune system toward Th2 responses. Clca3, Chi3l4, and Ear11 were the first lung biomarkers to increase in an asthma model and last biomarkers to decline in response to therapy; produced from alveolar macrophages after Th2 challenge	*(6,7)*
Fbp1	6		14121	*GO:0008152|metabolic process*<br>*GO:0005975|carbohydrate metabolic process*	A gluconeogenesis regulatory enzyme.	*None*
Mgl2	3-M2	4	216864		C-type lectin receptor: specifically recognizes N-acetylgalactosamine (GalNAc) and galactose. Up-regulated in M2-polarized mouse macrophages.	*(8-10)*
BC048546	6		232400	*GO:0010466|negative regulation of peptidase activity*	Proteinase inhibitor; unknown function; possible involvement with surfactant protein D immune complexes.	*(11)*
Gp2	1	3	67133		Transcytotic receptor for mucosal antigens (M cells); mondulates innate and adaptive immune responses in gut.	*(12,13)*
Ighv8-12	3		780960		Immunoglobulin heavy variable chain V8-12	*none*
Cxcr1	3		227288	*GO:0007186|G-protein coupled receptor signaling pathway*<br>*GO:0007165|signal transduction*<br>*GO:0007166|cell surface receptor signaling pathway*<br>*GO:0006935|chemotaxis*	Human homologue is receptor for interleukin-8; mediates and regulates inflammatory responses	*(14,15)*

Table 1 (Continued)

Ch25h	3-M2	5	12642	*GO:0008202|steroid metabolic process GO:0006633|fatty acid biosynthetic process GO:0055114|oxidation-reduction process GO:0006629|lipid metabolic process*	Cholesterol 25-hydroxylase; Component of regulatory network that modulates magnitude of innate immunity and intensity of subsequent adaptive responses. Produces oxysterols (immune regulators). Couples to Stat1 in macrophages.	*(16-18)*
Slc26a4	1	4	23985	*GO:0006820|anion transport GO:0006810|transport GO:0055085|transmembrane transport GO:0034220|ion transmembrane transport GO:0009887|organ morphogenesis GO:0032880|regulation of protein localization*	Pendrin; Cl^{-}/HCO^{-3} transporter; expressed in the bronchial epithelium following exposure to Th2 cytokines. Up-regulated in human asthma	*(19,20)*
Sncg	6		20618	*GO:0007268|synaptic transmission GO:0050808|synapse organization*	Involved in nerve function but also a cancer cell marker (prometastatic oncogene)	*(21)*
Kynu	3		70789	*GO:0008152|metabolic process*	Required for biosynthesis of NAD cofactors from tryptophan through the kynurenine pathway; Activation compensates for increased NAD demand during infection/inflammation.	*(22,23)*
Ptgir	3		19222	*GO:0007186|G-protein coupled receptor signaling pathway GO:0007165|signal transduction*	Prostacylin receptor; many functions related to inflammation and vascular responses.	*(24)*
Slc6a20a	6		102680	*GO:0006810|transport GO:0006865|amino acid transport*	Member of SLC6 Na^{+} and Cl^{-}dependent neurotransmitter transporter family.	*(25)*
Tnfrsf26	6		244237		Unknown function. No human homologue.	*UniGene Mm.247498 (26)*
Fetub	6		59083	*GO:0010466|negative regulation of peptidase activity GO:0010951|negative regulation of endopeptidase activity*	Cysteine protease inhibitor family member; poorly understood plasma protein.	*(27,28)*
Ttr			22139	*GO:0006810|transport*	Expressed as part of transgene.	*(29)*
Igk-V1	3		16081		Immunoglobulin kappa chain variable 1 (V1); ESTs exclusively in lung.	*UniGene Mm.304143 (30)*
Il7r	3		16197	*GO:0048872|homeostasis of number of cells GO:0000902|cell morphogenesis GO:0030217|T cell differentiation*	Alpha chain of IL7 receptor; IL-17 signaling has multiple roles in immune responses.	*(31,32)*
Mcoln3	6		171166	*GO:0006810|transport GO:0007626|locomotory behavior GO:0006811|ion transport*	Mucolipin: cation channel protein. Ca^{2+}-permeable channel; activity regulated by pH; crucial role in the regulation of cargo	*(33)*

					trafficking along endosomal pathway.	
Lilra5	3		232801		Crosslink of this receptor protein on monocytes induces proinflammatory cytokines, which suggests the roles in triggering innate immune responses; expressed on monocytes and neutrophils.	*(34)*
Cd200r4	3		239849		One of a family of receptors expressed on cells of myeloid and T-cell lineage; expression on the surface of airway macrophages induces a negative signaling cascade.	*(35)*
Tbxas1	3-M2	4	21391	*GO:0055114|oxidation-reduction process GO:0006631|fatty acid metabolic process GO:0006633|fatty acid biosynthetic process GO:0006629|lipid metabolic process*	Cytochrome P450 superfamily; converts prostaglandin H2 to thromboxane A2, a potent vasoconstrictor. Associated with M2 polarized macrophages and Th2 responses.	*(36,37)*
F7	3	4	14068	*GO:0007596|blood coagulation GO:0006508|proteolysis GO:0007599|hemostasis*	Coagulation cascade; role in Th2 responses in lung (eosinophilia, hyperresponsiveness, mucin production) have been demonstrated.	*(38)*
Arg1	3-M2	4	11846		Immune suppressor activity in myeloid derived cells. Stimulated by Th2 cytokines. M2 polarized macrophage marker.	*(39,40)*
Fabp1	5		14080	*GO:0006810|transport GO:0043066|negative regulation of apoptotic process*	Role in fatty acid uptake, transport, and metabolism.	*(41)*
Sult1d1	6		53315	*GO:0006629|lipid metabolic process*	No direct human homolog; catalyzes phenolic molecules; directly induced by glucocorticoids and may attenuate elevated catecholamine activity during stress.	*(42,43)*
Chst4	3		26887	*GO:0005975|carbohydrate metabolic process GO:0006954|inflammatory response*	Sulfotransferase that adds sulfates to L-selectin and signals homing of lymphocytes; increased during inflammatory stress.	*(44,45)*
Cyp2a4	6		13086		Cytochrome p450 member	
Thbs2	6		21826	*GO:0007155|cell adhesion*	Mediates cell-to-cell and cell-to matrix interactions; protective role against cardiac inflammation; upregulated by oxidative stress; antiangiogenic.	*(46-48)*
Myo5a	6		17918	*GO:0016192|vesicle-mediated transport GO:0050808|synapse organization GO:0007268|synaptic transmission*	Myosin protein involved in vesicle release	*(49)*
Syk	3		20963	*GO:0016310|phosphorylation GO:0007159|leukocyte cell-cell adhesion GO:0007166|cell surface receptor signaling pathway GO:0045087|innate immune response GO:0001525|angiogenesis*	Non-receptor type Tyr protein kinase; couples activated immunoreceptors to downstream signaling events; mediates proliferation, differentiation, and phagocytosis.	*(50)*

			GO:0006468|protein phosphorylation GO:0048514|blood vessel morphogenesis GO:0007167|enzyme linked receptor protein signaling pathway GO:0035556|intracellular signal transduction GO:0042742|defense response to bacterium GO:0007229|integrin-mediated signaling pathway GO:0050776|regulation of immune response GO:0007186|G-protein coupled receptor signaling pathway GO:0050715|positive regulation of cytokine secretion		
Dhcr7	5	13360	*GO:0030324|lung development GO:0030154|cell differentiation GO:0042127|regulation of cell proliferation GO:0001568|blood vessel development GO:0055114|oxidation-reduction process GO:0008202|steroid metabolic process GO:0006629|lipid metabolic process*	Enzyme that removes the C(7-8) double bond in the B ring of sterols and catalyzes the conversion of 7-dehydrocholesterol to cholesterol	*(51)*
Ly75	3	17076	*GO:0006897|endocytosis*	Mannose family receptor; expressed on antigen-presenting cell subsets, especially reported on dendritic cells. Homologous to macrophage mannose receptor.	*(52,53)*
Fnip2	6	329679	*GO:0001932|regulation of protein phosphorylation GO:0006468|protein phosphorylation*	Binds tumor suppressor gene folliculin and may be involved in apoptosis; mutations cause pulmonary cysts and pneumothorax.	*(54-56)*
Gp49a	3	14727		Cell surface antigen preferentially expressed on mouse mast cells	*(57)*
Abp1 (Aoc1)	6	76507		Actin binding protein implicated in cell movement	*(58)*
Cd177	3	68891		Neutrophil adhesion/migration molecule that binds to PECAM-1	*(59,60)*
Pigr	1	18703		Main receptor for transepithelial transport of IgA,the main Ig present on mucosal surfaces. Also regulates IgM export.	*(61,62)*
Mmp12	3-M2	17381	*GO:0006508|proteolysis*	Macrophage elastase; may play a role in emphysema and neutrophil influx. Preferentially expressed in M2 macrophages.	*(63,64)*
Chi3l4	1 | 3-M2 | 4	104183		Role in airway wall remodeling in the Th2-mediated allergic lung disease in mice.	*(65,66)*

Table 1 (Continued)

					Known to be expressed in dendritic cells in an IL-13-dependent manner.	
Clca3	1 2 4	23844	*GO:0006810|transport GO:0034220|ion transmembrane transport GO:0006811|ion transport*	Highly upregulated in Th2-epithelial mucus cell response. Biomarker for mucus cell metaplasia. See Ear11 above.	*(67,68)*
Itgax	3 4	16411	*GO:0007155|cell adhesion GO:0007229|integrin-mediated signaling pathway*	Combines with the beta 2 chain (ITGB2) to form a leukocyte-specific integrin One of main markers for dendritic cells. Necessary for Th2/Th17 responses in lung; primarily on myeloid cells.	*(69)*
Gpnmb	3	93695	*GO:0007155|cell adhesion*	Phagocytic protein involved in clearing apoptotic cells; known to act as a negative regulator of macrophage inflammatory responses.	*(70,71)*
Ctsk	3	13038	*GO:0045453|bone resorption GO:0006508|proteolysis*	Cysteine proteinase predominantly expressed in bone but also released from foamy (activated) macrophages.	*(72,73)*
Wdr16	6	71860		Reported in a screen for ciliated-cell specific genes.	*(74)*
Gla	6	11605	*GO:0008152|metabolic process GO:0005975|carbohydrate metabolic process*	Hydrolyses terminal alpha-galactosyl from glycolipids/glycoproteins. Deficiency leads to Fabry's disease; important for natural killer T cell function.	*(75,76)*
Lrp12	5	239393	*GO:0006897|endocytosis*	Related to low density lipoprotein receptor.	*(77)*
Csf2rb	3 4	12983		Beta chain for receptor for IL-3, IL-5 and CSF; defects associated with protein alveolar proteinosis; required for normal alveolar macrophage function; linked to Th2 inflammation	*(78,79)*
Lilrb4	3	14728		Receptor expressed on immune cells; binds to MHC class I molecules on antigen-presenting cells and transduces a negative signal that inhibits immune responses.	*(80-83)*
Mmp19	3	58223	*GO:0030154|cell differentiation GO:0006508|proteolysis GO:0001525|angiogenesis GO:0007275|multicellular organismal development*	Deficient mice show reduced cutaneous immune responses and altered T cell development; correlated with IL6 co-regulated proliferation genes; regulator of lung fibrosis.	*(84-86)*
Ms4a8a	3-M2	64381		CD20 homolog; expressed in macrophages; Induced with M2 macrophage mediators.	*(87,88)*
Slc39a2	3	214922	*GO:0072511|divalent inorganic cation transport*	Zinc transporter. Reported to be up-regulated in leukocytes of asthmatic infants.	*(89,90)*
Cd84	3	12523	*GO:0007155|cell adhesion*	Receptor involved in leukocyte activation; regulates macrophage cell-fate decisions and effector functions as well as T cell:B cell	*(91,92)*

Table 1 (Continued)

				contact and germinal center formation.	
Slc7a2	3	11988	*GO:0006810|transport GO:0015807|L-amino acid transport GO:0006865|amino acid transport GO:0006809|nitric oxide biosynthetic process*	Regulates arginine levels in macrophages	*(93,94)*
Bst1	3	12182		Also called CD157; GPI-anchored ADP-ribosyl cyclase,produces cyclic ADP-ribose from NAD(+) and acts as a receptor for adhesion and transmigration of monocytes.	*(95-97)*
Acp5	3	11433	*GO:0045453|bone resorption GO:0034097|response to cytokine stimulus*	Marker for bone osteoclasts/monocyte-derived cells; cleaved form is a potent ATPase; promotes cell motility. Mutations lead to autoimmune phenotypes. Important functions in immune defense.	*(98,99)*
Scgb1c1	3	338417		Secrotoglobulin found in upper airways and differentially regulated by cytokines	*(100)*
Mrc1	3-M2	17533	*GO:0007165|signal transduction GO:0006897|endocytosis*	Type I membrane receptor that mediates endocytosis of glycoproteins by macrophages; Marker of M2 polarized macrophages; also found on dendritic cells.	*(101-103)*
Rbp4	3 5	19662	*GO:0030324|lung development GO:0006810|transport*	Adipokine; known to be expressed in macrophages	*(104,105)*
Bcl2a1a	3	12044	*GO:0002903|negative regulation of B cell apoptotic process GO:0043066|negative regulation of apoptotic process GO:0001782|B cell homeostasis GO:0042981|regulation of apoptotic process GO:0006915|apoptotic process*	Reduces release of pro-apoptotic cytochrome c from mitochondria to block caspase activation. Direct transcription target of NF-kappa B, suggesting a cytoprotective function essential for lymphocyte activation and cell survival. Highly up-regulated in inflammatory macrophages.	*(106-108)*
Bcl2a1d	6	14121	*GO:0030217|T cell differentiation*	Anti-apopototic factor	*(109)*
Bcl2a1b	3	12045		See Bcl2a1a above	*(110)*
Ccl9	3	20308	*GO:0006955|immune response GO:0006935|chemotaxis*	Known to activate macrophages. Ligand for CCR1. Plays role in osteoclast differentiation and dendritic cell recruitment to Peyer's patches.	*(111-113)*
Anxa8	6	11752	*GO:0007596|blood coagulation GO:0007599|hemostasis*	Associated with terminal differentiation and actin structure as well as late endosome binding to actin.	*(114)*

Table 1 (Continued)

Chia	1	3-M2	4	81600	GO:0008152|metabolic process<br>GO:0006030|chitin metabolic process<br>GO:0090197|positive regulation of chemokine secretion<br>GO:0002532|production of molecular mediator involved in inflammatory response<br>GO:0006954|inflammatory response<br>GO:0006915|apoptotic process<br>GO:0006032|chitin catabolic process<br>GO:0005975|carbohydrate metabolic process	Chitinase induced by Th2/IL-13 pathways; expressed from both epithelial cells and macrophages.	(115-118)
Itgb2	3			16414	GO:0007159|leukocyte cell-cell adhesion<br>GO:0007229|integrin-mediated signaling pathway<br>GO:0016337|cell-cell adhesion<br>GO:0007155|cell adhesion<br>GO:0007275|multicellular organismal development	Integrin involved leukocyte cell adhesion. Defects cause leukocyte adhesion deficiency type I. Shown to be involved in macrophage efflux and local inflammatory control.	(119-121)
Clu	5			12759	GO:0043066|negative regulation of apoptotic process<br>GO:0008219|cell death<br>GO:0045597|positive regulation of cell differentiation<br>GO:0008284|positive regulation of cell proliferation	Clusterin, also known as apolipoprotein J; involved in multiple pathologies and biological processes	(122)
Cd68	3			12514	GO:0071310|cellular response to organic substance	Highly expressed by monocytes and tissue macrophages. Lectin-binding member of the scavenger receptor family.	(123,124)
Lipa	3-M2	5		16889	GO:0048771|tissue remodeling<br>GO:0030324|lung development<br>GO:0000902|cell morphogenesis<br>GO:0016042|lipid catabolic process<br>GO:0048873|homeostasis of number of cells within a tissue<br>GO:0001816|cytokine production<br>GO:0006954|inflammatory response<br>GO:0008283|cell proliferation<br>GO:0006631|fatty acid metabolic process<br>GO:0016125|sterol metabolic process<br>GO:0006629|lipid metabolic process	Catalyzes hydrolysis of cholesteryl esters/triglycerides; critical role in myeloid cell development and balance of immunosuppression and inflammation. Deficiency causes expansion of immature myeloid cells. Overexpression in transgenic mice polarizes macrophages to M2.	
H19	6			14955	GO:0010468|regulation of gene expression<br>GO:0008285|negative regulation of cell proliferation	Imprinted gene; non-coding RNA; encodes precursor of miR-675 in humans.	(130)
Chi3l3	1	3-M2	4	12655	GO:0006032|chitin catabolic process	Macrophage inflammatory response gene.	(131-135)

			GO:0005975|carbohydrate metabolic process<br>GO:0006954|inflammatory response	Marker of M2 polarized macrophages. See Ear11 above.	
Scnn1b		20277	GO:0006810|transport<br>GO:0006811|ion transport<br>GO:0050896|response to stimulus	Transgene	(29)
Hc	3	15139	GO:0006935|chemotaxis<br>GO:0045087|innate immune response<br>GO:0006954|inflammatory response<br>GO:0050921|positive regulation of chemotaxis	Complement C5. Involved in macrophage chemotaxis and cytokine production by macrophages.	(136-140)
Fn1	**3** **4**	14268	GO:0001525|angiogenesis<br>GO:0042060|wound healing<br>GO:0043066|negative regulation of apoptotic process<br>GO:0008360|regulation of cell shape<br>GO:0007160|cell-matrix adhesion<br>GO:0007155|cell adhesion<br>GO:0001775|cell activation<br>GO:0050921|positive regulation of chemotaxis	Fibronectin is involved in cell adhesion and migration processes including embryogenesis, wound healing, blood coagulation, host defense, and metastasis. Linked to Th2 mediated responses in monocyte-derived cells.	(36,141)
Ccl6	3 4	20305	GO:0060326|cell chemotaxis<br>GO:0006955|immune response<br>GO:0006935|chemotaxis	Plays a role in IL-13 induced inflammation and remodeling; chemoattractant for macrophages.	(142-144)
Reg3g	3	19695	GO:0006954|inflammatory response	Antibacterial C-type secreted lectin with activity against Gram-positive bacteria; antibacterial function for lung epithelium through Stat3-mediated induction	(145)
Retnla (Fizz1)	3-M2 4	57262		Macrophage M2-activation marker	(146-148)
Ctsd	3	13033	GO:0006508|proteolysis	Abundant macrophage lysosomal protease; regulates mitochondrial pathway of macrophage apoptosis or competing death processes, facilitating intracellular bacterial killing.	(149,150)

*Functional categories are as follows: 1) Epithelial Response to Stimulus; 2) mucus cell metaplastic response; 3) Immune response; (M2-activated macrophages designated as M2); 4) Link to Th2 inflammation 5) lipid/cholesterol metabolism; 6) Unknown functional link

Table 2. Percent Cell Counts (Percentage) on Purified Macrophage Fractions

Sample	Age (PND)	Genotype	Macrophages	Neutrophils	Eosinophils	Lymphocytes
1*	0	WT	98.05	0.00	0.00	1.95
2*	0	WT	97.49	0.00	0.00	2.51
3*	0	WT	98.88	0.00	0.00	1.12
4*	0	Tg	98.36	0.00	0.00	1.64
5*	0	Tg	98.99	0.00	0.00	1.01
6*	0	Tg	99.04	0.00	0.00	0.96
7	3	WT	94.30	0.00	0.00	5.70
8	3	WT	95.76	0.00	0.00	4.24
9	3	WT	97.24	0.00	0.00	2.76
10	3	WT	96.51	0.00	0.00	3.49
11	3	Tg	98.50	0.00	0.00	1.50
12	3	Tg	93.68	2.11	0.00	4.21
13	3	Tg	93.78	1.55	1.55	3.11
14#	3	Tg	-	-	-	-
15	10	WT	98.68	0.00	0.00	1.32
16	10	WT	98.43	0.00	0.00	1.57
17	10	WT	98.32	0.00	0.00	1.68
18	10	WT	99.45	0.00	0.00	0.55
19	10	Tg	92.74	2.23	3.35	1.68
20#	10	Tg	-	-	-	-
21	10	Tg	94.15	0.00	0.00	5.85
22	10	Tg	96.24	1.61	2.15	0.00
23	42	WT	97.37	0.00	0.38	2.26
24	42	WT	97.69	0.00	0.00	2.31
25	42	WT	96.56	0.38	0.00	3.05
26	42	WT	93.43	0.73	0.00	5.84
27	42	Tg	94.14	0.00	1.17	4.69
28	42	Tg	95.80	0.38	1.91	1.91
29	42	Tg	93.77	0.35	1.73	4.15
30	42	Tg	95.37	0.00	0.71	3.91
31	42-GF	WT	96.07	0.00	0.00	3.93
32	42-GF	WT	97.22	0.00	0.00	2.78
33	42-GF	WT	97.10	0.00	0.00	2.90
34	42-GF	Tg	94.84	0.40	1.59	3.17
35	42-GF	Tg	96.68	0.00	0.83	2.49
36	42-GF	Tg	95.11	1.13	2.63	1.13

*** Differential cell counts were performed on Pre-column BAL samples.**
# Poor quality of cytospin staining prevented accurate differential cell counting.

Table 3. Top cytokine signaling gene level table corresponding to the "Cytokine Signaling" Gene Ontology pathway results for differential gene expression in whole lung and macrophages comparing *Scnn1b*-Tg mice to WT at the time points indicated. Genes are grouped into categories based upon the characteristics defined in the headings. Genes are only shown if the fold-change was ±2-fold for any condition. Light red indicates up-regulation in *Scnn1b*-Tg compared to WT. Green indicates down-regulation.

	Fold-changes in Whole Lung				Fold-changes in Macrophages			
**Gene Symbol**	**PND0**	**PND3**	**PND10**	**PND42**	**PND0**	**PND3**	**PND10**	**PND42**
**Differentially regulated in lung only, never macrophages**								
Tlr2	-1.10	1.07	2.18	1.06	-1.10	1.53	1.24	-1.48
Clec4n	1.03	1.09	2.32	1.76	-1.17	1.51	1.55	-1.44
Cxcl5	1.29	2.89	5.10	1.91	-1.05	1.00	-1.06	1.04
Ltf	-1.62	1.15	1.95	3.75	1.62	1.76	1.06	1.10
Chia	-1.14	-1.20	-1.31	3.49	1.15	1.11	1.08	1.02
Clec7a	1.03	-1.11	1.47	2.65	-1.04	-1.01	1.09	1.20
Lipa	-1.27	1.07	1.13	2.42	-1.22	-1.04	1.27	1.36
Acp5	-1.02	1.00	1.67	2.10	-1.35	1.09	1.32	1.80
Hc	1.06	-1.10	-1.26	2.05	-1.63	-1.03	1.03	1.03
Syk	-1.01	1.10	1.24	2.03	-1.30	1.26	1.14	1.18
**Mixed lung and macrophage signatures**								
Ccl3	1.04	2.38	10.82	12.21	-1.49	5.79	5.68	13.16
Clec4e	1.02	1.39	5.52	1.10	-1.66	3.99	12.34	1.05
Irg1	1.19	1.22	2.09	1.41	-1.00	13.90	17.13	2.75
Slc11a1	-1.07	-1.05	2.03	1.60	-1.21	8.78	12.82	6.20
Cd14	1.02	1.33	2.65	1.94	-1.05	4.66	3.42	-1.61
Pglyrp1	-1.64	1.16	2.20	2.08	1.15	3.08	-2.58	3.22
Chi3l1	-1.20	1.05	1.22	2.20	-1.08	20.56	1.76	-1.05
Clec5a	-1.08	-1.21	3.27	2.74	-1.19	1.77	1.71	2.19
Tnfrsf9	1.09	-1.01	1.57	2.49	-1.11	3.01	1.55	1.40
**Differentially Regulated by PND 0 in macrophages**								
Rsad2	-1.63	-1.75	-1.11	-1.10	15.95	-1.09	1.33	-1.13
Apoa1	-1.52	-1.18	1.09	-1.08	8.30	1.04	1.18	-1.08
Pf4	1.05	1.62	1.20	-1.06	4.61	4.25	1.93	2.67

Supplemental Table 3 (Continued)

Cd59a	-1.22	-1.06	1.02	1.02	3.60	1.18	1.31	1.36
Cd34	-1.26	-1.19	-1.17	-1.00	3.58	-1.28	-1.47	1.27
Snai2	-1.06	-1.42	-1.16	-1.20	3.33	1.01	1.00	1.08
Hdac7	1.21	1.12	-1.08	1.04	2.77	1.14	-1.04	1.01
Ndrg2	-1.00	1.38	-1.03	-1.46	2.67	1.07	1.04	-1.08
Apoa1	-1.02	1.01	1.02	1.19	2.61	-1.01	1.01	-1.04
Adipoq	-1.02	1.08	-1.08	1.17	2.57	1.05	1.10	-1.04
Tgfb2	-1.00	-1.25	-1.19	-1.14	2.52	1.23	-1.08	-3.19
Figf	-1.37	-1.45	-1.03	1.32	2.41	1.06	-1.05	1.08
Ptgs2	-1.21	1.16	1.55	-1.51	2.38	11.96	1.89	1.29
Irf7	-1.31	-1.27	1.19	1.06	2.22	1.59	1.18	-1.08
Agtr2	-1.18	1.24	1.38	1.00	2.20	-1.04	-1.05	1.05
Cidea	1.03	1.03	1.01	1.00	2.18	-1.01	1.00	-1.02
Prnp	-1.23	1.02	-1.03	1.08	2.12	2.42	1.22	3.08
Pawr	-1.12	1.13	1.16	1.01	2.12	1.04	1.06	1.02
Smad3	1.06	1.09	-1.20	1.01	2.11	2.22	-1.02	1.17
Afap1l2	-1.05	-1.20	-1.22	-1.13	2.09	-1.01	1.00	-1.03
S1pr3	-1.15	-1.31	-1.10	1.01	2.08	-1.02	-1.07	-1.07
Ffar4	1.22	1.64	1.66	-1.20	2.06	-2.52	-1.55	-1.06
Irak4	1.13	-1.07	-1.03	1.11	-2.03	-1.27	-1.03	-1.18
Nlrc4	-1.01	-1.02	1.03	1.18	-2.04	-1.36	-1.17	-1.40
**Differentially regulated by PND3 in macrophages**								
Thbs1	-1.14	-1.07	-1.42	1.40	1.50	27.30	3.91	2.43
Ccl4	1.11	-1.19	1.12	1.23	1.13	12.04	4.20	5.22
Adam8	-1.05	1.58	1.36	1.88	1.12	9.27	1.84	3.91
Ccl2	1.15	-1.33	1.03	1.20	-1.28	8.05	2.94	7.50
Il1f9	-1.00	-1.27	1.53	1.24	-1.14	7.90	2.63	1.46
Hilpda	-1.08	-1.06	-1.16	-1.09	-1.16	7.78	5.85	1.41
Htr2b	1.05	1.04	1.12	1.16	1.35	6.60	1.74	1.20
Il1b	-1.08	1.27	1.63	1.32	-1.55	5.78	3.03	3.13
C3ar1	-1.12	-1.02	1.69	1.63	-1.58	5.37	2.46	7.44

Ereg	-1.11	-1.21	-1.03	1.00	-1.05	5.00	1.82	-1.06
Cmklr1	-1.03	1.09	-1.06	1.04	1.33	4.33	1.07	1.27
Srgn	-1.19	-1.06	1.06	1.36	-1.16	3.88	2.07	2.30
Ptafr	-1.07	-1.20	1.47	1.43	-1.23	3.68	2.34	4.01
Il10	-1.02	1.02	-1.10	1.05	-1.01	3.62	1.03	-1.02
Abca1	-1.07	1.28	-1.16	1.42	-1.04	3.52	2.54	-1.36
Tnfaip3	1.09	1.06	1.10	1.23	-1.02	3.17	1.45	1.16
Mefv	1.07	-1.03	1.00	1.04	-1.04	3.08	-1.01	1.06
Adora2b	-1.00	1.11	1.16	1.38	-1.16	3.07	1.67	1.19
Tnf	1.05	-1.18	1.36	1.08	-1.34	2.86	3.31	1.28
Irak3	-1.00	-1.05	1.42	1.43	-1.06	2.85	1.64	-1.05
Tlr1	1.01	-1.04	1.00	1.20	-1.08	2.80	1.35	1.22
Il6	1.02	-1.29	1.05	-1.08	-1.02	2.73	1.29	1.26
Trem3	-1.37	-1.57	1.04	1.04	-1.12	2.72	1.29	-1.69
Malt1	1.22	1.01	1.42	1.69	-1.10	2.54	2.59	1.53
Src	-1.07	1.14	-1.01	-1.08	1.80	2.39	1.05	1.50
Il1a	1.15	-1.25	2.05	1.22	-1.04	2.33	1.85	2.63
Fcgr2b	-1.17	-1.04	1.30	1.90	-1.23	2.33	1.36	1.32
Tlr9	1.01	-1.00	1.08	1.22	-1.04	2.29	1.16	1.06
Cd74	-1.22	-1.85	-1.15	1.49	-1.25	2.07	-1.03	1.18
Runx3	1.03	-1.01	1.03	1.03	1.06	2.03	1.09	1.79
Rbpj	1.06	-1.16	-1.15	1.01	-1.02	2.03	1.32	1.22
Klf2	-1.07	-1.04	-1.03	1.10	1.03	2.01	1.06	-1.05
Gadd45g	-1.13	1.13	1.03	1.55	-1.35	-2.04	-1.84	1.41
Ccr2	1.10	-1.46	-1.01	1.27	-1.05	-2.15	-1.20	1.01
**Differentially regulated by PND10 in macrophages**								
Rnf128	-1.19	1.38	1.51	1.57	1.24	3.42	5.45	5.09
Il20rb	1.08	1.03	-1.07	1.15	-1.33	1.99	2.70	1.87
Clec9a	1.04	1.10	-1.03	1.39	1.00	1.29	2.46	2.73
Cd276	-1.03	-1.00	-1.06	1.07	-1.02	1.43	2.22	1.04
Il13	1.16	-1.07	-1.09	1.06	-1.09	-1.09	-2.31	4.62

Table 3 (Continued)

Prg2	-1.02	1.05	-1.15	1.06	-1.02	1.05	-2.39	3.79
**Differentially regulated at PND42 only in macrophages**								
Hpse	-1.27	1.14	1.24	1.24	1.63	1.12	1.57	11.44
Rgcc	-1.15	1.83	1.31	1.27	-1.44	1.42	1.29	9.76
Arrb1	1.10	-1.02	-1.19	-1.11	1.26	1.09	1.40	5.32
Scgb1a1	-1.87	-1.72	-1.54	-1.05	1.18	-1.63	1.45	3.86
Fabp4	1.01	1.11	1.19	1.57	-1.31	-1.06	1.23	3.19
Ccr5	1.01	-1.07	1.13	1.59	-1.48	1.80	-1.15	2.81
Scamp5	-1.06	-1.02	-1.00	1.05	1.02	-1.01	1.41	2.36
Ccr7	-1.01	-1.06	-1.18	1.05	1.16	1.01	-1.25	2.33
Cebpe	1.09	-1.01	-1.07	1.09	-1.24	1.23	-1.89	2.10
Il1rl1	-1.04	-1.04	-1.20	1.27	1.00	1.03	-1.10	2.04
Rnf19b	-1.05	-1.04	1.08	1.52	-1.34	1.98	1.29	2.00
Zbtb32	1.10	1.01	1.09	1.16	-1.79	-1.27	-1.63	-2.23
Trem1	-1.60	-1.06	1.14	-1.03	-1.27	1.54	1.02	-2.30
