# Supplementary figures and images for: Gene expression in whole lung and pulmonary macrophages reflects the dynamic pathology associated with airway surface dehydration
Source: BMC Genomics. 2014 Sep 10;15(1):726. doi: 10.1186/1471-2164-15-726 (PMC4247008; doi:10.1186/1471-2164-15-726)

**a**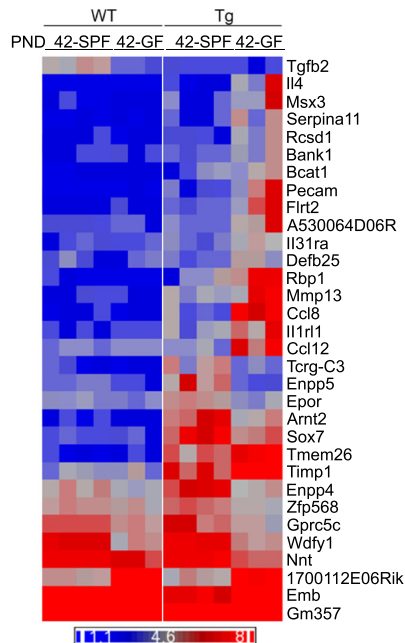**b**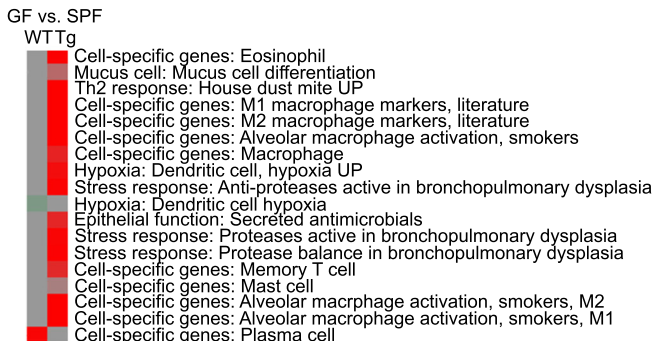**c**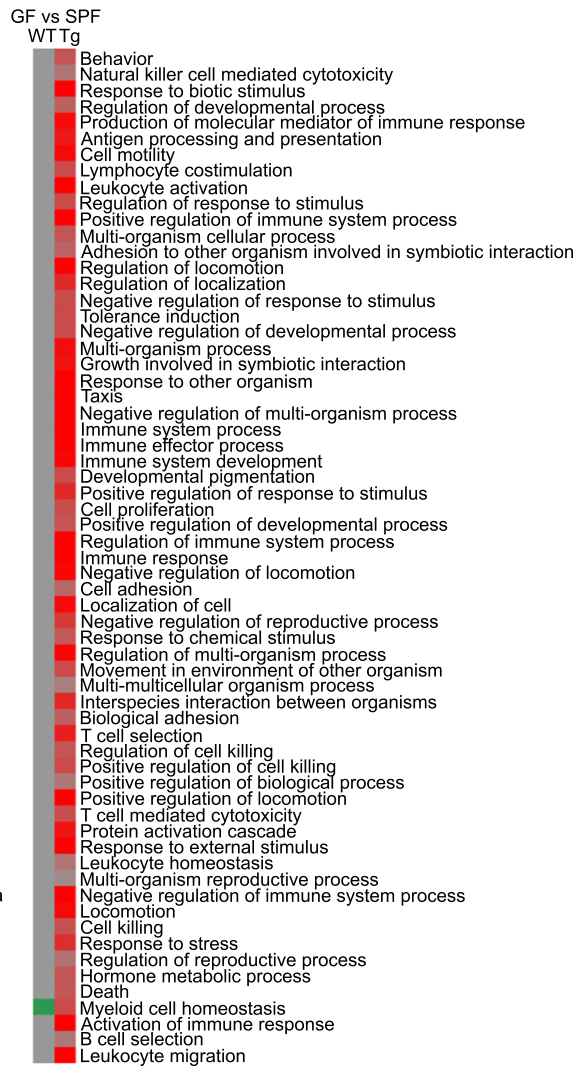

Supplement: Supplementary file 7 — Additional file 7: Results file S5: This excel file contains data tables for Figure 6. For each comparison between Scnn1b-Tg vs WT, GSEA enrichment summary tables (consult http://www.broadinstitute.org/gsea/ documentation for output format) GO biological process terms branching off from "Cytokine production" were copied into each spreadsheet table as indicated in the table name. To regenerate the results, GO terms were displayed as connected networks by Cytoscape ( http://www.cytoscape.org/) and the FDR values were used to convert to enrichment scores described above, from each GSEA summary table saved as tab-delimited text files were used to decorate the GO term nodes as color intensities. The fold change values for "Cytokine production" genes were filtered at minimal of 1.5 fold change between Scnn1b-Tg and WT mice for at least 1 time point, then used for clustering in Additional file 1: Figure S6 are in "CytoGene_fold_change_1.5" worksheet. (PDF 781 KB) [file 12864_2014_6683_MOESM7_ESM.pdf]
